# Toward Two-Dimensional van der Waals Magnon Transport Devices: WTe_2_ Electrodes for Efficient Magnon Spin Injection and Detection

**DOI:** 10.1021/acsnano.5c14307

**Published:** 2025-10-25

**Authors:** Krishnaraajan Sundararajan, Dennis K. de Wal, Sergio Alvarruiz, Cédric A. Cordero-Silis, Majid Ahmadi, Marcos H. D. Guimarães, Bart J. van Wees

**Affiliations:** Zernike Institute for Advanced Materials, 3647 University of Groningen, NL-9747 AG Groningen, The Netherlands

**Keywords:** van der Waals, nonlocal magnon transport, antiferromagnets, spin Hall effect, unconventional charge-to-spin interconversion

## Abstract

One of the bottlenecks toward all two-dimensional material-based magnon transport devices is the absence of a two-dimensional material for the efficient injection and detection of magnon spins. Here, we demonstrate that WTe_2_, a layered, nonmagnetic van der Waals material, functions as an efficient spin injector and detector for magnon spins. It enables injection and detection of spins polarized in-plane via the conventional spin Hall effect and magnon spins polarized out-of-plane through unconventional charge-to-spin interconversion mechanisms. Such dual functionality is not achievable with conventional electrodes such as platinum or permalloy in the absence of a magnetic field. Using CrPS_4_, an insulating two-dimensional antiferromagnet, we employ a hybrid nonlocal device geometry where magnon spins are injected and detected via conventional platinum and WTe_2_ contacts. We find that the effective charge-to-spin conversion efficiency of WTe_2_ is about 0.45 and 1.7 times for the injection of in- and out-of-plane polarized spins, respectively, compared to the in-plane polarized spin injection efficiency of platinum electrodes.

## Graphical abstract

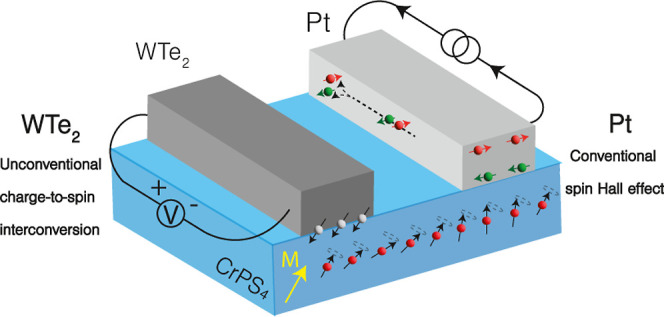

1

The study of collective excitations of magnetic order, spin waves or their quanta magnons, has attracted significant research attention as magnon-based spin currents offer a promising alternative as information carriers for low-power and high-speed wave-based computation.^1^ For practical application of magnons, electrically controlled magnon spin transport^2–4^ proves to be an attractive and promising approach and has been investigated in a wide range of magnetic systems ranging from ferrimagnetic oxides,^2,3^ antiferromagnetic oxides,^5–7^ and in van der Waals materials.^8,9^ Two-dimensional (2D) van der Waals materials provide a rich platform to reveal the fundamental magnon spin transport properties owing to their versatility such as various magnetic textures,^10–12^ easy scalability down to nanometer scale,^12^ manipulation of the magnetic ground state in van der Waals heterostructures,^13^ and potential control of magnetic properties via electric fields.^14^

In nonlocal magnon spin transport experiments, platinum (Pt) is used as the conventional electrode to inject and detect magnon spins via the spin Hall effect (SHE) and inverse SHE, respectively.^2,3,5–9^ Although relatively efficient, Pt has the disadvantage that the conventional SHE in Pt results in spins polarized only in-plane. Electrodes that inject out-of-plane polarized spins are essential for studying spin-flop dynamics in systems such as CrPS_4_ (Figure 1a), which can offer insight into how magnon transport evolves across magnetic phase transitions. Although recent nonlocal spin Seebeck (nonlocal SSE) measurements employing Pt under a tilted magnetic field have been able to detect the spin-flop transition in CrPS_4_,^15^ the impact of this transition on nonlocal magnon transport is barely explored, as existing experiments utilize the conventional SHE in Pt.

**Figure 1:**
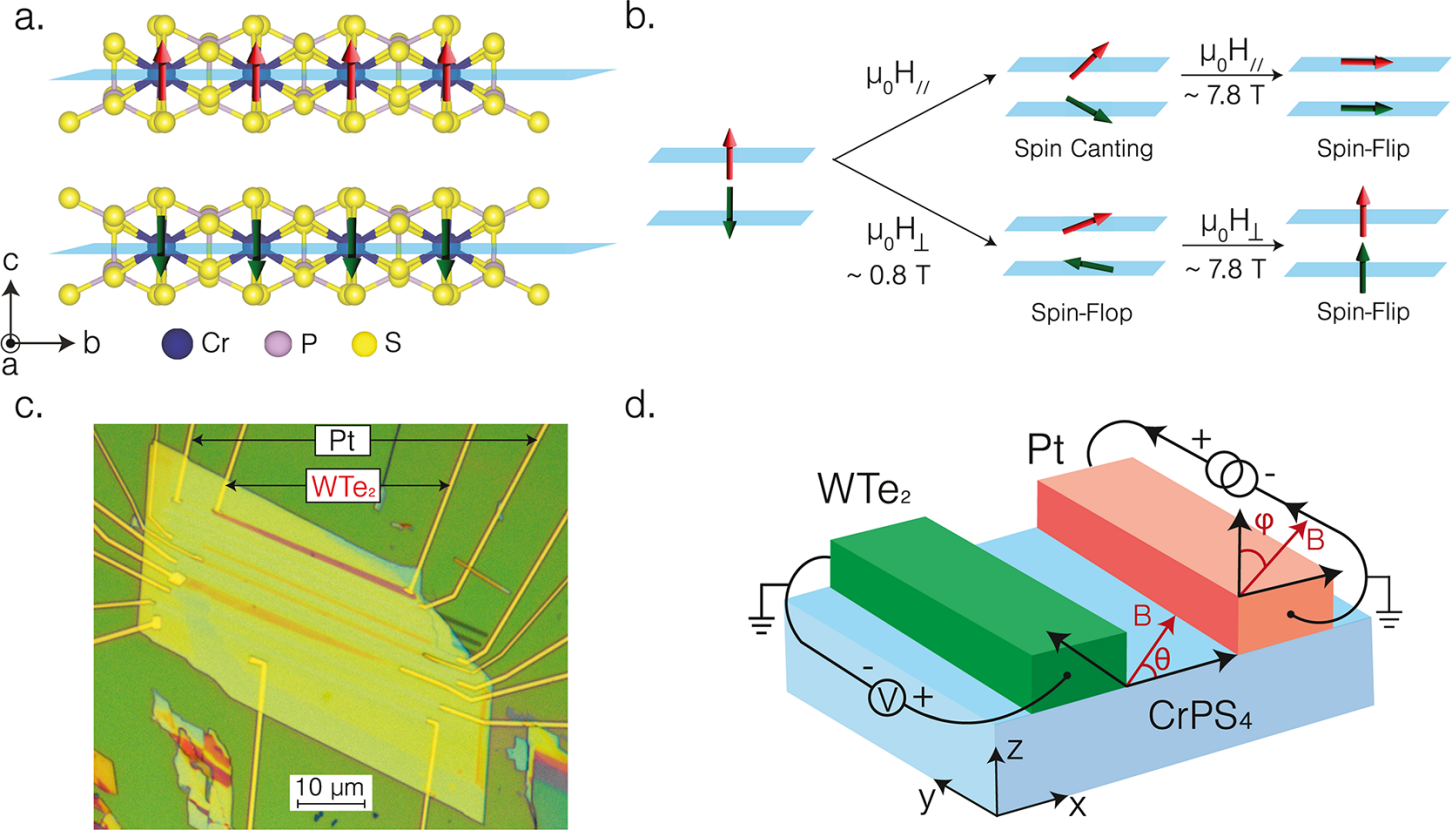
(a) Crystal structure of CrPS_4_, where the red/blue arrows on the Cr^3+^ ions illustrate their magnetic moments displaying A-type antiferromagnetic ordering, (b) an illustration of the magnetic phase transition of CrPS_4_ under an applied external magnetic field, (c) optical microscope image of the device with WTe_2_ and Pt electrodes on CrPS_4_, and (d) illustration of the nonlocal measurement geometry employing Pt and WTe_2_ electrodes with the angle definitions, with θ being the in-plane magnetic field angle and φ being the out-of-plane magnetic field angle.

Permalloy (Py) has enabled the injection of out-of-plane polarized spins in yttrium iron garnet (YIG) via the anomalous SHE^16^ and detection of the spin-flop transition in MnPS_3_.^17^ The main drawback of this approach is that Py contacts exhibit a large shape anisotropy and require large external magnetic fields (∼1 T) to overcome this. Additionally, Pt and Py are typically deposited via DC sputtering. In systems like YIG, it has been shown that thermal annealing of the substrate prior to metal deposition significantly enhances the interface quality.^18^ However, for van der Waals magnets, sputtering metal contacts tend to produce an amorphous interface, as observed at the Pt/MnPS_3_ interface^19^ and similarly in our experiments with CrPS_4_ (see Section XI of the Supporting Information for scanning/transmission electron microscopy (TEM) of the interface). This method of electrode deposition therefore leads to invasive contacts that disrupt the surface integrity of the two-dimensional material. Although evaporated Pt and Py contacts offer a less invasive alternative, they typically exhibit a lower interfacial spin mixing conductance^20^ or fail to inject magnonic spin currents electrically, as reported for CrBr_3_.^21^ These limitations prevent reliable injection and detection of magnon spins in van der Waals magnets approaching the 2D limit, making 2D magnon spin injectors essential to probe magnon transport down to the monolayer limit. Furthermore, an electrode that can efficiently inject and detect both in-plane and out-of-plane polarized magnon spin opens new possibilities for studying magnon transport in arbitrarily magnetized systems (including spin-flop transitions). Such electrodes are also crucial for observing effects that rely on out-of-plane magnon spin injection such as the magnon Hall effect.^22^

In contrast to metal contacts, van der Waals materials can be stacked into heterostructures with atomically clean interfaces with a high transparency for spin injection. Among the family of van der Waals materials, WTe_2_ is a promising candidate and has been extensively studied for its electronic and spin transport properties. Notable features include its extremely large nonsaturating magnetoresistance (MR),^23^ significant thermoelectric power,^24,25^ temperature-induced Lifshitz transition,^26^ and the onset of superconductivity under applied external pressure.^27^ Owing to its sizable spin–orbit coupling, WTe_2_ has been investigated as a topological Weyl semimetal candidate^28^ and for its low-symmetry-aided generation of an out-of-plane antidamping torque.^29–31^ Recent electronic spin transport measurements have additionally shown the potential of WTe_2_ for its charge-to-spin interconversion at room temperature due to both conventional SHE and unconventional charge-to-spin interconversion.^32,33^

In WTe_2_, the conventional SHE for an in-plane charge current produces a transverse spin current flowing out-of-plane, with the spins polarized in a direction perpendicular to both the charge and spin current directions, similar to that for Pt. Furthermore, studies have revealed that the spin Hall conductivity in WTe_2_ exhibits a marked dependence on the direction of the applied charge current, differing significantly when the current flows along the crystallographic *a*-axis versus the *b*-axis.^34,35^ Additional charge-to-spin interconversion also produces a spin polarization along the *c*-axis, normal to the WTe_2_ interface when a charge current is applied along the crystallographic *a*-axis. Although the symmetry constraints of bulk WTe_2_ forbid such a spin polarization direction,^36^ it has been experimentally observed and has been attributed to the breaking of glide mirror symmetry (mirror reflection followed by translation) at the interface for thin WTe_2_ flakes,^29^ but has also been observed in substantially thicker flakes as well, up to 16 nm using second harmonic Hall measurements in a WTe_2_/Py heterostructure.^31^

Here, as a proof of concept, we report the electrical injection and detection of both in-plane and out-of-plane polarized magnon spins in van der Waals antiferromagnet CrPS_4_ utilizing the conventional and unconventional charge-to-spin interconversion of WTe_2_. Using a hybrid device geometry that incorporates WTe_2_ and Pt electrodes, we isolate and investigate the role of WTe_2_ in the injection and detection of magnon spins. As magnon transport in CrPS_4_ has been widely studied,^8,9^ it serves as an ideal reference system for the study of magnon spin injection utilizing another van der Waals material. Although the electronic spin transport of WTe_2_ has been investigated via spin transfer torque ferromagnetic resonance (FMR) measurements utilizing a WTe_2_/Py heterostructure^29,31^ or in a nonlocal device geometry,^33,37^ the advantage of employing a nonlocal magnon transport geometry in comparison to the nonlocal electronic geometries reported in ref 33 is that the charge current in the circuit lies completely in-plane in the injector strip and is free of parasitic leakage current paths and facilitates the study of charge-to-spin interconversion (and vice versa) of WTe_2_ without a local charge current in WTe_2_.

## 2 Results and Discussion

In this study, we employ CrPS_4_—an A-type antiferromagnetic insulator—as our magnon transport medium (Figure 1a), in which Cr^3+^ ions couple ferromagnetically within each layer, as shown in Figure 1a. Under an out-of-plane magnetic field (*H*_⊥_//*ĉ*) of ∼0.8 T, CrPS_4_ undergoes a spin-flop transition and around 8T fully aligns into a fully saturated collinear magnetic state, as illustrated in Figure 1b. The measurement device used in this work is shown in Figure 1c,d, which shows an illustration of the nonlocal measurement geometry (see Supporting Information III for details on electronic-to-magnon spin interconversion and the effect of charge-to-spin interconversion mechanisms on nonlocal magnon transport). The device consists of a 25 nm WTe_2_ strip (1.15 μm wide, 30 μm long) and a 12 nm thick Pt strip (675 nm wide, 40 μm long) on a 56 nm thick CrPS_4_ (for SEM and AFM, see Supporting Information I.A). Polarized Raman spectroscopy was used to confirm that the WTe_2_ electrode was defined at an angle of −12° with respect to its crystallographic *a*-axis (see Supporting Information I.B). For this study, all the devices were fabricated with the strip oriented along the crystallographic *a*-axis of WTe_2_, as based on previous reports, we expect out-of-plane polarized spins only for charge current along the crystallographic *a*-axis.^29–31,33^ For electrical measurements, an alternating-current (ac) of 3.33 Hz was used in the injector strip. With an ac current, the electrically generated and detected magnons and those detected as a result of a thermal gradient due to Joule heating of the injector can be decoupled with a lock-in measurement by measuring the first and second harmonic responses, respectively. During measurements, the magnetization of CrPS_4_ was monitored utilizing the local Spin Seebeck Effect (local SSE) across Pt (for spin Hall magnetoresistance across Pt, see Supporting Information VII).

By rotating the magnetic field direction in-plane (θ) or in the out-of-plane direction (φ) as illustrated in Figure 1c, the magnetization of CrPS_4_ is changed and thus the polarization of the magnon spins, which is antiparallel to the magnetization. Figure 2a,b illustrates the expected nonlocal resistance for the hybrid geometry used. The different possible polarizations of the spin accumulation at the WTe_2_/CrPS_4_ interface are illustrated with $\sigma_{WTe_{2}}^{x,y,z}$ , where $\sigma_{WTe_{2}}^{x}$ corresponds to the conventional SHE. The nonlocal voltage is a combination of both the injection and detection of magnon spins and is proportional to the projection of the polarization of the spin accumulation of the injector and detector onto the magnetization direction. For out-of-plane angular rotations (along the *xz*-plane) of the magnetic field, the unconventional charge-to-spin conversion in WTe_2_, namely, the spins polarized along *ẑ* $\left(\sigma_{WTe_{2}}^{z}\right)$ , enables WTe_2_ to be sensitive to magnon spins polarized along *ẑ* and $\sigma_{WTe_{2}}^{x}$ to the magnon spins polarized in-plane. Platinum, owing to the conventional SHE, is only sensitive to spins polarized in-plane along the interface. The combination of injecting in-plane polarized spins and detecting out-of-plane polarized spins (or vice versa) results in a maximum of the nonlocal voltage when the magnetization has equal projections both in-plane and out-of-plane, i.e., at φ_0_ = 45°. Similarly, for in-plane angular rotation, the effect of the charge-to-spin interconversion of WTe_2_ is shown in Figure 2b. We stress that the measurement geometry is sensitive to unconventional charge-to-spin conversion processes in WTe_2_. Distinct charge-to-spin conversion processes cause a measurable angular phase shift of the nonlocal voltage response during magnetic field rotations. Tracking this shift allows us to distinguish between different spin polarization contributions.

**Figure 2:**
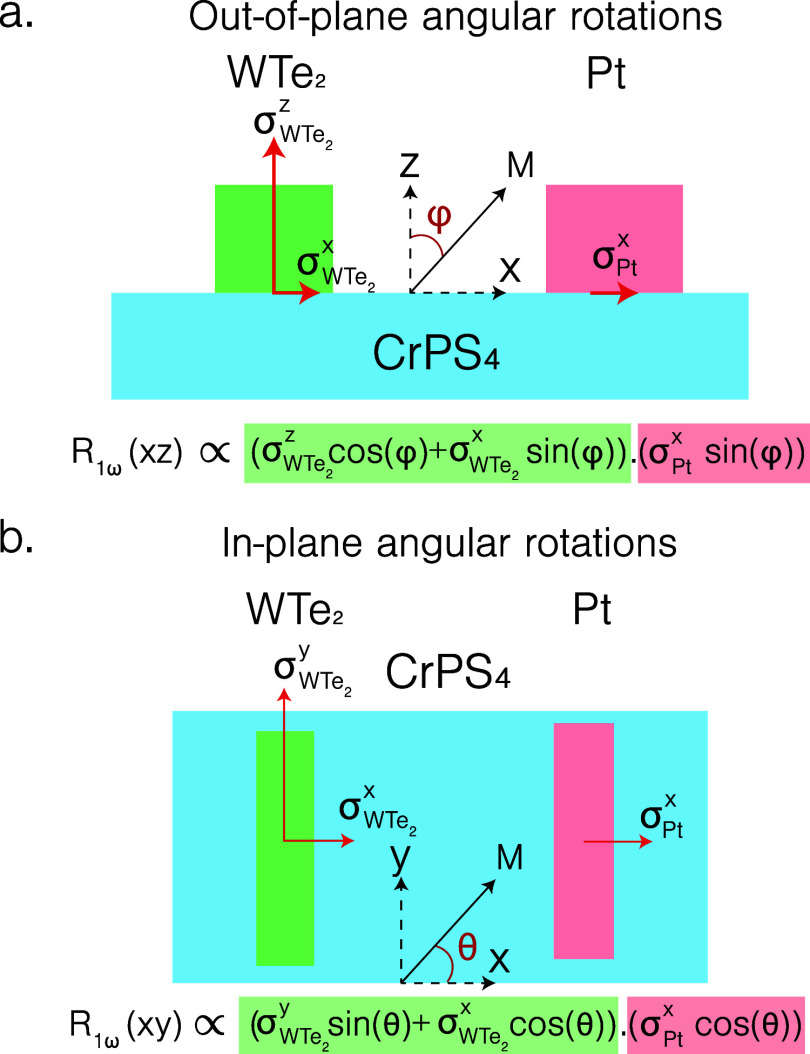
Illustration of the expected nonlocal resistance arising due to different charge-to-spin interconversion processes for (a) out-of-plane angular rotations and (b) in-plane angular rotations of the magnetic field.

For the device geometry, for WTe_2_, assuming that only one mirror symmetry plane along the *bc*-plane exists, we expect contributions only from $\sigma_{WTe_{2}}^{x}$ (corresponding to the conventional SHE) and $\sigma_{WTe_{2}}^{z}$ (due to the unconventional charge-to-spin interconversion process). We expect no contributions from $\sigma_{WTe_{2}}^{y}$ that result in spins polarized along the strip, as the crystal symmetry forbids it. We thus expect the nonlocal resistance to be

1 $$R_{1\omega \left(xz\right)}^{NL}\propto \frac{\sigma_{WTe_{2}}^{z}\sigma_{Pt}^{x}}{2}\times \sin\left(2\varphi \right)+\sigma_{WTe_{2}}^{x}\sigma_{Pt}^{x}\sin^{2}\left(\varphi \right)$$

2 $$R_{1\omega \left(xy\right)}^{NL}\propto \sigma_{WTe_{2}}^{x}\sigma_{Pt}^{x}\cos^{2}\left(\theta \right)$$

where *R*_1ω(*xz*)_^NL^ and *R*_1ω(*xy*)_^NL^ denote the first harmonic nonlocal response as a function of out-of-plane and in-plane magnetic field rotation, respectively. This is obtained from the projection of the polarizations of the spin accumulation of the injector and the detector onto the magnetization, as illustrated in Figure 2a,b.

We exclude parasitic effects such as leakage charge current, parasitic capacitive coupling, MR of WTe_2_, and the magnetotransport properties of CrPS_4_ that could give rise to the nonlocal responses (for details, see Supporting Information II). We emphasize that unlike Pt electrodes, where MR effects are negligible, WTe_2_ exhibits a pronounced MR response under an out-of-plane magnetic field. This introduces additional complexity in the interpretation of nonlocal measurements. Specifically, the intrinsic MR of WTe_2_, combined with capacitive coupling between electrodes, can give rise to spurious nonlocal signals originating from instrumental cross-talk. As such, careful experimental design and data analysis are required to reliably isolate and extract the magnonic contribution (for details of the data analysis, see Supporting Information V). The local second harmonic response arising from local SSE and the nonlocal first harmonic response are fit with the periodic functions (for details of fitting and removal of outliers, see Supporting Information IV)

3 $$R_{2\omega }^{Loc}=\frac{V_{2\omega }^{Loc}}{I^{2}}=\Delta R_{2\omega }^{Loc}\;\sin\left(\alpha +\alpha_{Loc}\right)+R_{2\omega }^{offset}$$

4 $$R_{1\omega }^{NL}=\frac{V_{1\omega }^{NL}}{I}=\Delta R_{1\omega }^{NL}\sin^{2}\left(\alpha +\alpha_{NL}\right)+R_{1\omega }^{offset}$$

where α = θ for in-plane angular rotations and φ for out-of-plane angular rotations, α_Loc/NL_ is the angular phase shift of the response, and Δ*R*_2ω_^Loc^ and Δ*R*_1ω_^NL^ correspond to the amplitudes of the local second harmonic and nonlocal first harmonic resistances, respectively. We emphasize that α_Loc/NL_ captures the charge-to-spin interconversion process. For instance, for the combination of injecting in-plane and detecting out-of-plane polarized spins, we expect a 45° angular phase shift between the local SSE (α_Loc_ = 0°) and the nonlocal first harmonic voltage (α_Loc_ = 45°) for the out-of-plane angular rotations. On the contrary, in the case where both electrodes are Pt, out-of-plane magnetic field rotation results in the observed angular phase shifts of α_Loc_ = 0° and α_NL_ = 0°.^9^

The local second harmonic across Pt and the nonlocal resistance across the WTe_2_ detector as a function of the in-plane magnetic field angle are shown in Figure 3a. The measurements are obtained at an applied magnetic field of 8T at 25 K, which corresponds to a fully saturated collinear magnetic state of CrPS_4_. By comparing the angular phase shift of the nonlocal signal to that of the local SSE measured across Pt, we find that the signal is dominated by the conventional SHE of WTe_2_ (as for the case of Pt), with no additional contribution from unconventional charge-to-spin interconversion mechanisms that would result in spins polarized along the charge current direction, as expected from symmetry constraints. Furthermore, comparing the signs of the nonlocal first harmonic response (positive/negative) to the local SSE, we conclude that Pt and WTe_2_ have the same sign of spin Hall angle for a charge current applied along the crystallographic *a*-axis (offset by −12°; for details, see Supporting Information III).

**Figure 3:**
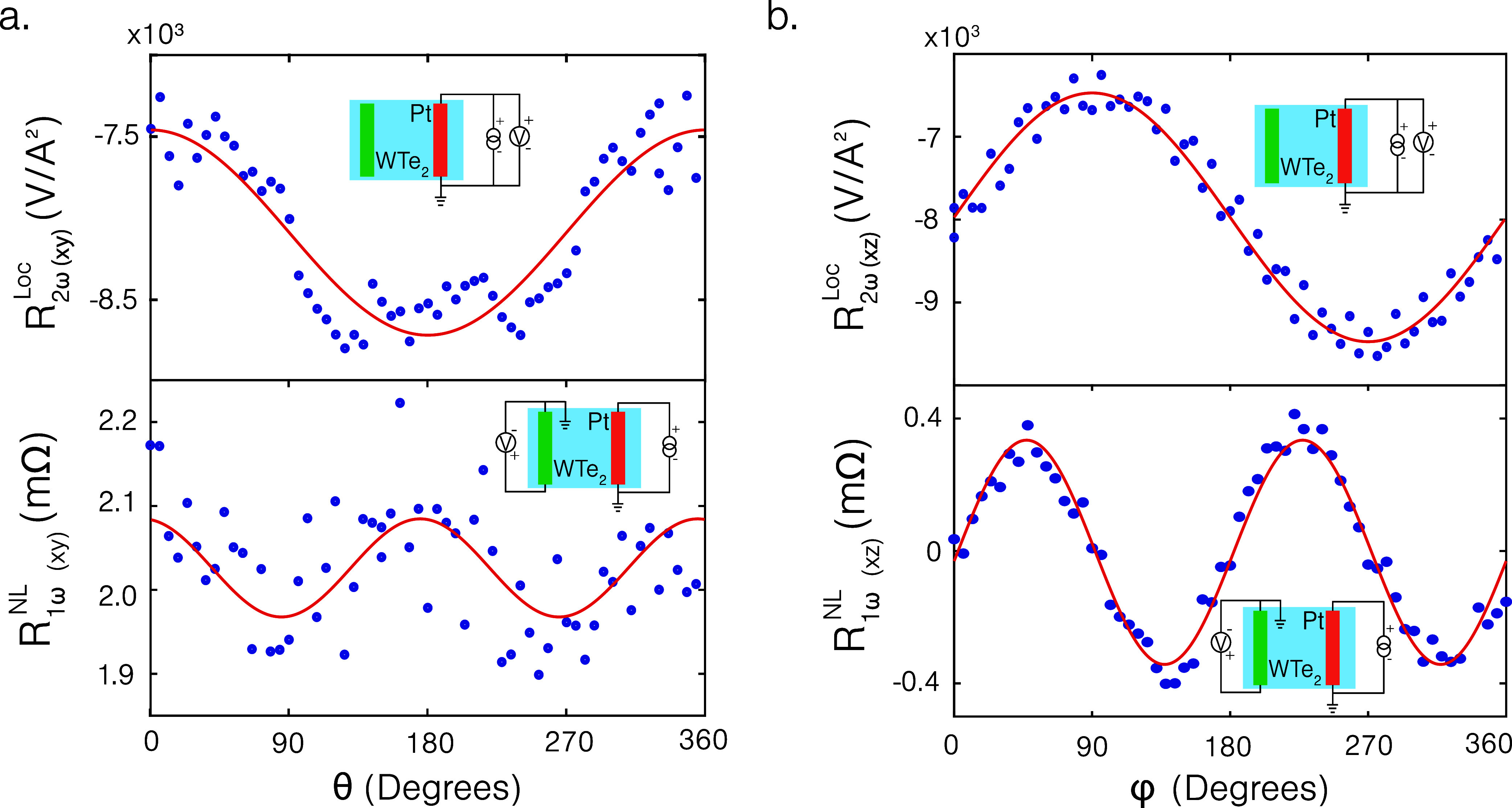
Local second harmonic response across Pt (local SSE) and the nonlocal first harmonic response for in-plane magnetic field rotations (at 25 K, 8T) and (b) the local second harmonic response across Pt and the nonlocal first harmonic response for out-of-plane magnetic field rotations (at 15 K, 8T).

To explore the unconventional charge-to-spin interconversion of WTe_2_ for magnon spin detection, we performed out-of-plane angular rotations. We observe a maximum at φ = 45° in the extracted nonlocal first harmonic response (for details of the analysis of the out-of-plane angular rotations, see Supporting Information V), as shown in Figure 3b, indicating that one of the electrodes is sensitive to out-of-plane polarized spins. In the case of Pt as both injector and detector electrodes, no nonlocal signal for out-of-plane magnetic fields was observed.^9^ We thus attribute this angular phase shift to the polarization of spins generated by Pt lying in-plane perpendicular to the strip and the detection of magnon spins by WTe_2_ to be sensitive along the out-of-plane direction. We note that the nonlocal signal arising due to the in-plane polarized spins detected by WTe_2_ could not be extracted separately from the out-of-plane angular scans due to the process of extracting the contribution of the out-of-plane polarized spins (for details, see Supporting Information V). Furthermore, we note that the bias current applied, 100 μA, is within the linear response of the system and the nonlocal voltage is completely reciprocal upon switching the injector–detector combination obeying reciprocity (see Supporting Information VI).

The amplitude of the nonlocal resistance modulation (Δ*R*_1ω(*xy*)_^NL^ as defined in eq 4) for the in-plane applied magnetic field, shown in Figure 4a, is consistent with previous reports using only Pt electrodes.^8,9^ We observe a sharp increase in the nonlocal signal around 8T, close to the spin-flip field of CrPS_4_. The signal also shows a characteristic increase in the nonlocal signal around 25 K, which has been attributed to a combined effect of temperature on the saturation magnetization of CrPS_4_ and the reduction in the equilibrium magnon density and the magnon conductivity at lower temperatures.^8^

**Figure 4:**
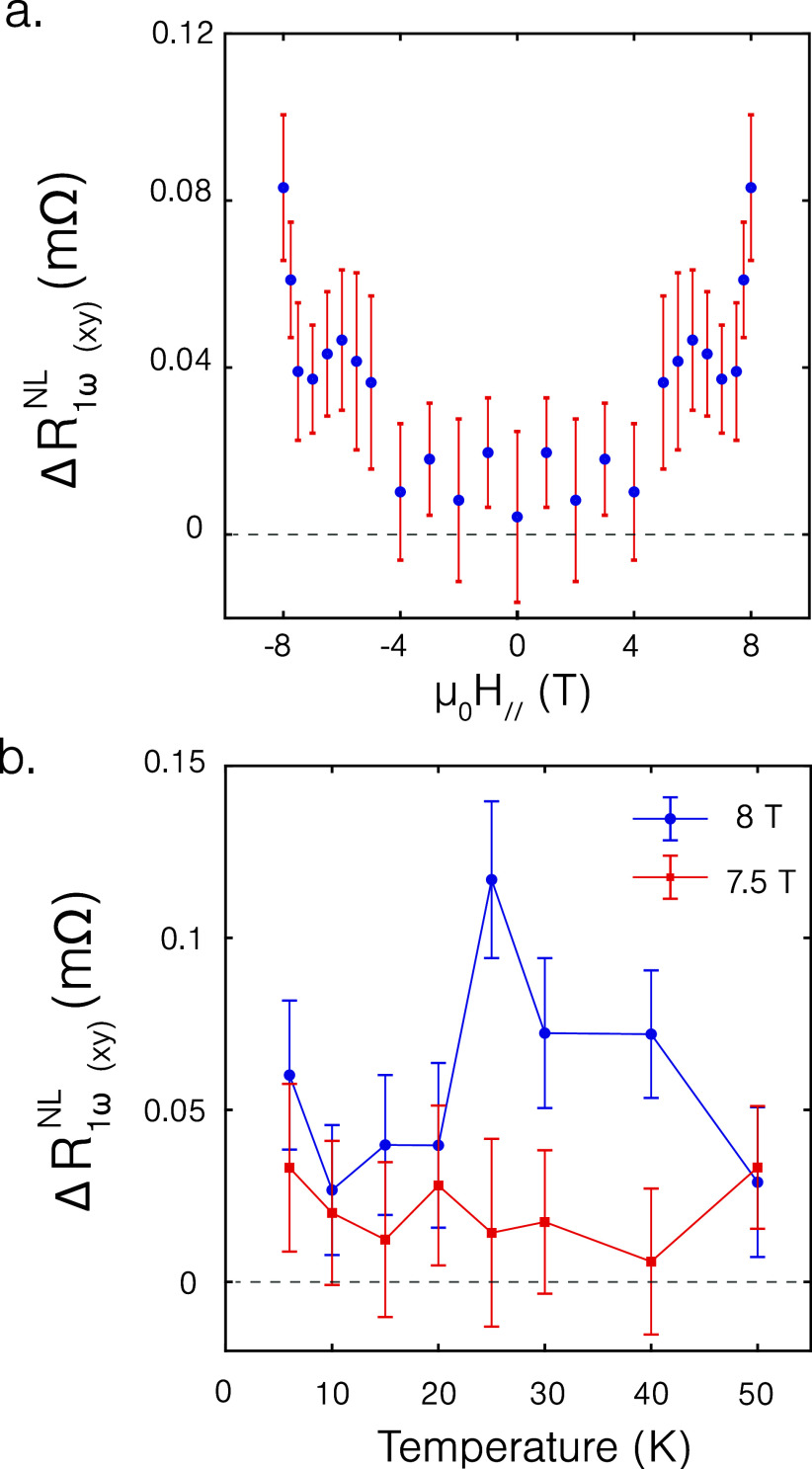
Amplitude of the nonlocal resistance modulation while injecting with Pt and detecting with WTe_2_ as a function of (a) applied external in-plane magnetic field at 25 K and (b) temperature for in-plane angular rotations (amplitude without symmetrization).

The amplitude of the nonlocal resistance modulation (Δ*R*_1ω(*xz*)_^NL^ as defined in eq 4) as a function of magnetic field at 25 K for out-of-plane angular rotations is shown in Figure 5a. We observe that for the transport of out-of-plane polarized magnon spins, a trend similar to that of the in-plane polarized magnon spins is observed, namely, no detectable transport until a collinear magnetization configuration is reached, suggesting similar magnon transport properties for in- and out-of-plane magnetized system. Furthermore, measurements on an additional sample (see Supporting Information X) exhibit saturation in the nonlocal signal for the transport of out-of-plane polarized magnon spins, similar to ref 9 upon saturation of the magnetization, allowing us to conclude that the charge-to-spin interconversion in WTe_2_ is not influenced by an external magnetic field.

**Figure 5:**
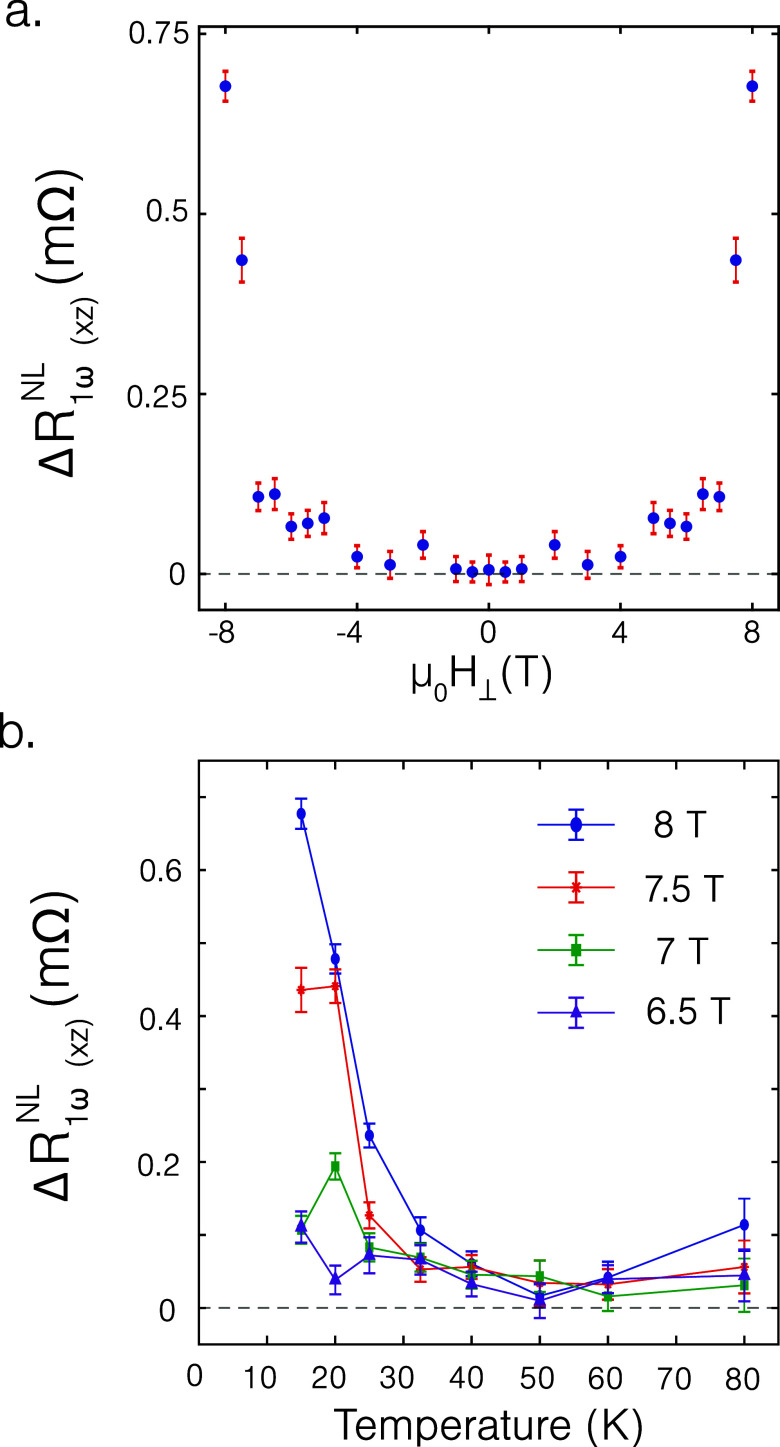
Amplitude of the nonlocal resistance modulation while injecting with Pt and detecting with WTe_2_ as a function of (a) applied external out-of-plane magnetic field at 15 K and (b) temperature for out-of-plane angular rotations.

The temperature dependence of the amplitude of the nonlocal resistance modulation for the transport of out-of-plane polarized magnon spins is shown in Figure 5b, where we observe an increase in the nonlocal signal below the Néel temperature of CrPS_4_ (38 K for bulk CrPS_4_ ^38^). Surprisingly, we observe an increase in the nonlocal resistance modulation for an applied field of 8T for 15 K in comparison to 20 K unlike for the in-plane polarized magnon spins. Possible explanations for this trend could be due to a different temperature dependence of the magnon transport properties for magnon spins polarized out-of-plane in comparison to in-plane polarized spins or due to a possible temperature dependence of the charge-to-spin interconversion process in WTe_2_. Further investigation of the magnon conductivity of CrPS_4_ as a function of temperature for both in- and out-of-plane polarized magnon spins is necessary to further understand this trend.

To quantify the magnon injection efficiency, we adopt the injector conversion coefficient defined in refs 3 and 39 , hereafter referred to as the charge-to-spin conversion efficiency (see Supporting Information IX for details). From the amplitude of the nonlocal resistance modulation, we estimate the effective (a combination of the charge-to-spin interconversion and the interfacial spin-mixing conductance, for details, see Supporting Information IX) injection/detection of charge-to-spin conversion efficiency of WTe_2_ for in-plane and out-of-plane polarized spins as 0.03 and 0.12 Ω, respectively. Conventional Pt electrodes (300 nm wide and 7 nm thick) have an efficiency of 0.07 Ω for the injection of in-plane polarized spins (for details, see Supporting Information IX). Comparing these values, we find that WTe_2_ exhibits about 0.5 and 1.7 times the efficiency for injection of in- and out-of-plane polarized spins in comparison to the in-plane spin injection efficiency of Pt. We note that the extraction of the effective spin mixing conductance at the WTe_2_/CrPS_4_ interface is not possible as the spin Hall magnetoresistance (SMR) across WTe_2_ is overwhelmed by its MR, and similarly, the local SSE on WTe_2_ is overwhelmed by the bilinear magnetoresistance. Despite observing a magnetization-dependent nonlocal second harmonic response of WTe_2_, the nonlocal SSE of WTe_2_ is overshadowed by the large Nernst effect of WTe_2_ (for details, see Supporting Information VIII).

Spin-transfer torque measurements on WTe_2_/Py heterostructures have consistently shown higher efficiency for in-plane polarized spins over out-of-plane polarized spins.^29,31^ However, in our magnon spin transport experiments, we find the out-of-plane polarized magnon spin transport to be more effective. Possible explanations could be anisotropic spin mixing conductance/spin diffusion length for in- and out-of-plane polarized spins. Furthermore, recent studies on NbIrTe_4_, which possesses the same bulk crystal symmetry, reveal a transition in which the out-of-plane spin transfer torque becomes dominant beyond a critical thickness, highlighting a dimensionality-driven crossover.^40^ A detailed investigation of the thickness dependence of WTe_2_ may provide deeper insight into the dimensionality effects governing the magnon spin injection.

We also studied the geometry with both the injector and the detector as WTe_2_, and due to the large MR and capacitive cross-talk, separate extraction of the magnon transport signal was not possible (for details, see Supporting Information XII). However, WTe_2_ offers a promising platform for injection and detection of arbitrarily polarized magnon spins for magnets with lower saturation fields where the MR of WTe_2_ is smaller. Cross-sectional TEM analysis reveals that CrPS_4_ oxidizes under ambient atmospheric conditions forming an amorphous CrO_*x*_ oxide layer (see Supporting Information XI). Interestingly, this oxide layer is absent at the Pt/CrPS_4_ interface, likely due to oxide removal during Pt sputtering. Further EDX analysis shows an ∼1 nm intermediate PtS_*x*_ layer at the Pt/CrPS_4_ interface. At the WTe_2_/CrPS_4_ interface, a distinct amorphous TeO_*x*_ layer is observed, indicating that WTe_2_ reacts with the native CrO_*x*_ layer. Furthermore, we observe this layer to have recessed below the pristine CrPS_4_ surface, indicating tellurium diffusion into the CrO_*x*_-rich region. While these oxide layers may impact efficiency, the persistence of the transport signals suggests that magnon transport remains active and offers a clear path to improve the device performance by means of interface engineering to reduce oxidation and amorphous interface formation at the interface.

## 3 Conclusion

In summary, we report the ability of WTe_2_, a nonmagnetic van der Waals material to inject and detect magnon spins by utilizing CrPS_4_ as the magnon transport medium. We observe injection and detection of magnon spins consistent with the conventional spin Hall effect for in-plane polarized magnon spins and additionally the detection of out-of-plane polarized magnon spins owing to the unconventional charge-to-spin interconversion in WTe_2_. Unlike ferromagnetic Py for injection of out-of-plane polarized spins, WTe_2_ does not require an externally applied magnetic field, making it a more versatile candidate for out-of-plane polarized magnon spin injection. While the exact interfacial spin mixing conductance could not be determined, the modulation of nonlocal resistance for out-of-plane magnon spins suggests efficient charge-to-spin conversion and good interfacial spin transparency in WTe_2_, comparable to sputtered Pt. Furthermore, our results of integration of two-dimensional materials as spin injectors open up avenues for potentially addressing the magnon modes of air-unstable 2D magnets electrically in an all two-dimensional magnon transport device, where the earlier approaches of depositing Pt were proving experimentally incompatible. Although we do not observe features associated with the spin-flop of CrPS_4_, a magnon spin injector and detector sensitive to the out-of-plane polarized spins provide new possibilities for the detection of the spin-flop transition. This also highlights the need for more effective low-crystal-symmetry materials such as TaIrTe_4_ ^41,42^ and NbIrTe_4_ ^40^ as alternatives for the injection and detection of magnon spins. Additionally, the possibility for injection and detection of out-of-plane polarized magnon spins should allow for electrically probing the magnon Hall effect.^22^

## 4 Methods

### 4.1 Experimental Methods

Bulk CrPS_4_ and WTe_2_ crystals were purchased from HQ Graphene. CrPS_4_ flakes were exfoliated onto a Si/SiO_2_ (285 nm) substrate under an ambient atmosphere using the standard scotch-tape method. Using electron beam lithography on the CrPS_4_ flake, strips were defined, and platinum (Pt) (∼12 nm) was deposited by DC sputtering. The measured device was fabricated by dry transfer^43^ of a WTe_2_ flake (∼25 nm, initially selected by means of optical contrast) onto the CrPS_4_ flake (∼56 nm) with the predefined Pt strips in a glovebox under inert conditions (N_2_ glovebox, <0.1 ppm of O_2_, <0.5 ppm of H_2_O). The WTe_2_ was then etched into strips by reactive ion etching with CF_4_/O_2_ employing a poly­(methyl methacrylate) (PMMA) mask defined using electron beam lithography. Before the deposition of the Ti/Au (5/70 nm) contacts onto WTe_2_ and Pt strips, WTe_2_ was cleaned in situ by using argon ion milling to remove any residual oxide layer at the top interface. The final sample was spin-coated with PMMA to prevent the WTe_2_ strip from further oxidation.

### 4.2 Electrical Transport Measurements

The various electrical measurements at different temperatures were carried out by varying the magnetic field or by rotating the sample with respect to the applied magnetic field in a superconducting solenoid cryostat (Cryogenic Limited). An alternating current of 100 μA was sourced at a frequency of 3.333 Hz (unless otherwise mentioned), and the voltage was measured by standard lock-in techniques (SR830 and AMETEK 7270). For in-plane angular rotations, a 29° sample-rotator offset was corrected (for details, see Supporting Information IV) as estimated from the LSSE response.

## Supplementary Material



## Notes

Further details of device fabrication and characterization (AFM, polarized Raman spectroscopy, and cross-sectional TEM), details on exclusion of parasitic contributions to the nonlocal signal, details on the relation between the angular phase shift and the charge-to-spin interconversion mechanism, details of data processing, details of excluding the magnetoresistance effect of WTe_2_ in the measured nonlocal signal, calculation of interfacial spin conversion efficiency, and electrical measurements of two additional devices (PDF)
