# Supplementary material for: Toward Two-Dimensional van der Waals Magnon Transport Devices: WTe2 Electrodes for Efficient Magnon Spin Injection and Detection
Source: ACS Nano. 2025 Oct 25;19(44):38716–23. doi: 10.1021/acsnano.5c14307 (PMC12613838; doi:10.1021/acsnano.5c14307)
Supplement: Supplementary file 1 [file nn5c14307_si_001.pdf]

Supplemental materials to " Towards two-dimensional van der Waals magnon transport devices: WTe_2 electrodes for efficient magnon spin injection and detection"

Krishnaraajan Sundararajan,[1] Dennis K. de Wal,[1] Sergio Alvarruiz,[1] Cédric A. Cordero-Silis,[1] Majid Ahmadi,[1] Marcos H.D. Guimarães,[1] and Bart J.van Wees[1]

[1] *Zernike Institute for Advanced Materials, University of Groningen, NL-9747 AG Groningen, The Netherlands*

Contents

I. Device Characterization 3
I.A. Atomic Force Microscope (AFM) and Scanning Electron Microscopy (SEM) of the device 4
I.B. Polarized Raman Measurements of the device 6

II. Excluding Parasitic Contributions to the Non-Local Resistance 7
II.A. Excluding Magnetoconductance of $CrPS_4$ 7
II.B. Excluding Parasitic Leakage Current 7

III. Expected Non-Local response due to magnon transport 9

IV. Data processing and fitting procedure 11
IV.A. Symmetrization and Anti-Symmetrization of Data 11
IV.B. Fitting Procedure and Error bar estimation 11
IV.C. Removal of Rotator Offset 11

V. Analysis of out-of-plane field rotations 12
V.A. Magnetoresistance of WTe_2 12
V.B. Effect of MR of WTe_2 on the non-local voltage 13
V.C. Decoupling parasitic effect of the MR from the detected non-local voltage 15

VI. Verification of Linear Response and Reciprocity 19

VII. Spin Hall Magnetoresistance (SMR) of Platinum 20

VIII. Analysis of Second Harmonic Responses 21
VIII.A. Local Second Harmonic response of WTe_2 21
VIII.B. Non-Local Second Harmonic response of WTe_2 22
VIII.C. Non-Local Second Harmonic response of Pt 23

IX. Comparison of interface spin conversion efficiency 24

X. Comparison with Device S1 25

XI. Cross-Sectional Transmission Electron Microscope 26

XII. Comparison with Device S2 30

References 32

I. DEVICE CHARACTERIZATION

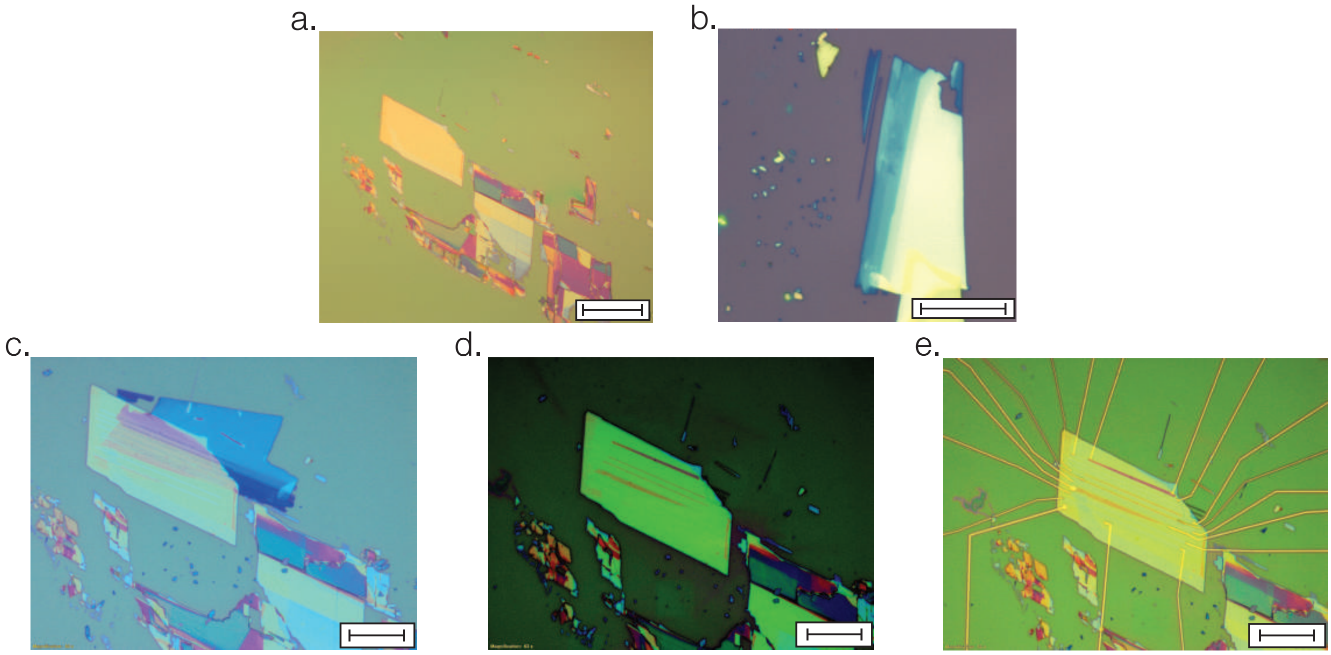

FIG. S1: Optical microscope images of the device fabrication process: a) the $CrPS_4$ flake, b) WTe_2 flake, c) the $WTe_2/CrPS_4$ (with Pt deposited via sputtering) hetrostructure, d) after etching of the WTe_2 flake and e) the final device with Ti/Au contacts. (Scale Bar: 20 μm)

I.A. Atomic Force Microscope (AFM) and Scanning Electron Microscopy (SEM) of the device

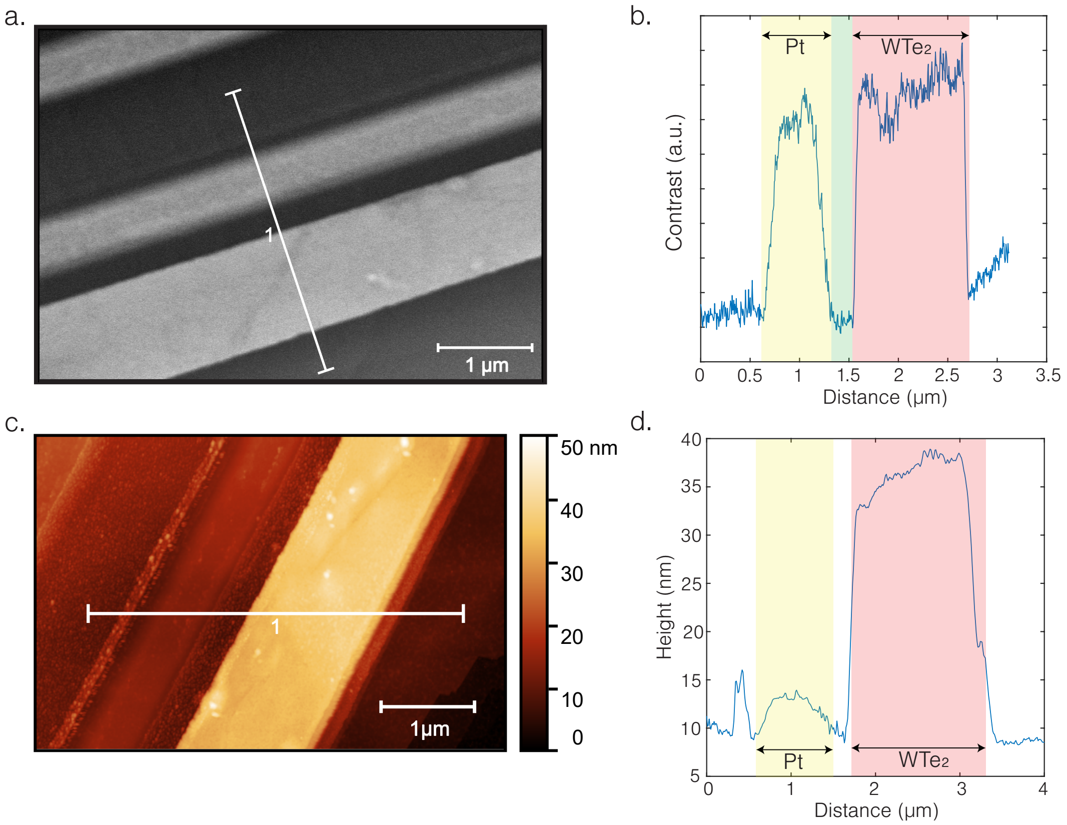

FIG. S2: a) Scanning Electron Micrograph of the device, b) width profile obtained from the SEM image, c) Atomic Force Micrograph of the device, d) height profile obtained from the AFM image.

The SEM and AFM micrographs of the final device are shown in Fig. S2a,c respectively. The width of the Pt and WTe_2 strips were estimated from SEM to be 675 nm and 1.15 μm respectively, while the distance between the strips is estimated as 220 nm (edge-to-edge). Furthermore, the lengths of the Pt and WTe_2 strips are approximated from SEM as 40 μm and 30 μm respectively. The thickness of the flakes are estimated as 25 nm for WTe_2 and 56 nm for $CrPS_4$ using AFM. The AFM and the SEM micrographs were obtained after the electrical characterization of the sample, in the absence of the PMMA protective layer.

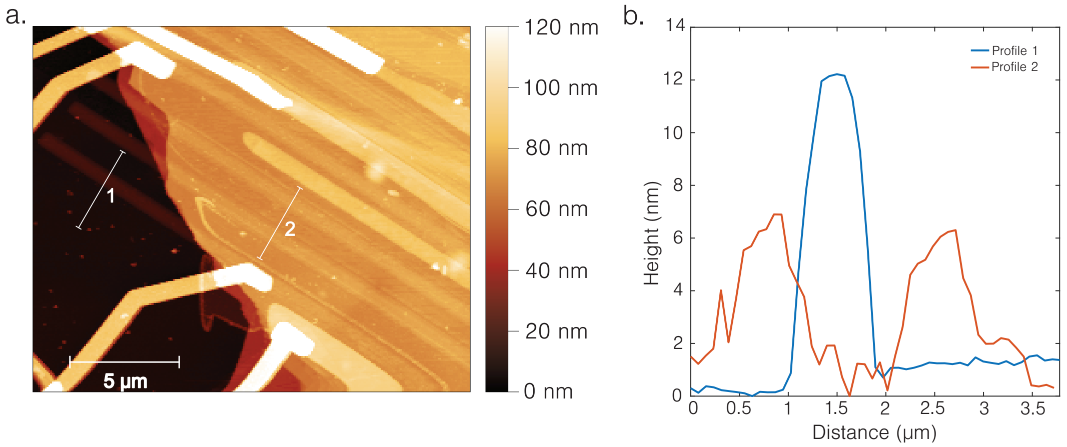

FIG. S3: a) Atomic Force Micrograph of the final device highlighting Pt deposited on the Si/SiO_2 substrate and on $CrPS_4$, b) The height profiles corresponding to profile 1 and 2 in (a).

Further AFM analysis reveals that depositing Pt contacts onto $CrPS_4$ through DC sputtering is abrasive to the surface of the two-dimensional magnet. The AFM of the measured device is shown in Fig. S3a, where two different profiles of Pt are highlighted: profile 1 corresponds to Pt sputtered on the Si/SiO_2 substrate and profile 2 corresponds to Pt sputtered on $CrPS_4$. The process of Pt deposition was achieved in the same step of DC sputtering and from the height profiles shown in Fig. S3b we observe a height difference of ~ 5 nm. Further characterization of the Pt/$CrPS_4$ interface was performed using transmission electron microscopy (TEM) and is discussed later (see section SI XI).

I.B. Polarized Raman Measurements of the device

Because of its low-symmetry lattice packing, WTe_2 possesses vibrational modes whose Raman tensor elements differ along various crystallographic directions. The crystal axes of the WTe_2 and $CrPS_4$ flakes used in the device fabrication (device in the main text) are determined from polarized Raman measurements based on previously reported analogous measurements [1, 2].

The spectra are obtained with an inVia Raman Renishaw microscope using a linearly polarized laser in back-scattering parallel geometry. The excitation wavelength and the grating used were $\lambda = 532$ nm and 1800 l/mm, respectively. The laser power was ∼100 µW with a diffraction-limited spot of ∼ 1µm positioned on one of the WTe_2 strips and on the $CrPS_4$ area of the finished device. The sample was rotated in steps of ∼10° for each measurement and optical images were recorded before each measurement to determine the exact rotation.

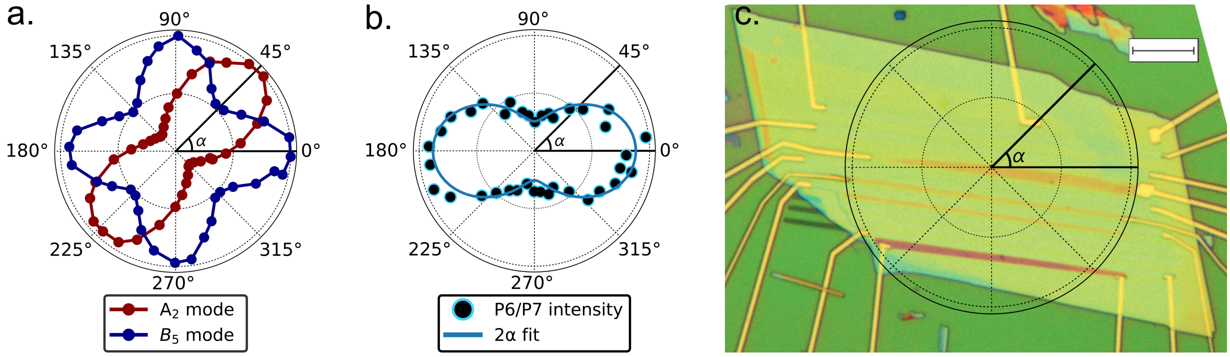

FIG. S4: Polarized Raman spectroscopy for crystal axis determination, a) Bare $CrPS_4$ polarization dependence of the bands at 169 cm^{-1} (A_2 mode), characteristic of the b-axis at 532 nm excitation, and 256 cm^{-1} (B_5 mode), b) Polarization dependence of the WTe_2 measured at the bottom most strip (from device shown in C). The ratio of peaks P6 and P7 is used to determine the a-axis as described in the text, c) Optical microscope image of the device used for the Raman measurements (Scale Bar: 10 μm). For all subfigures, α denotes the laser polarization angle.

For $CrPS_4$, the peaks at ∼169 cm^{-1} and ∼256 cm^{-1} are singly degenerate vibrational modes and for the modes at 532 nm the 169 cm^{-1} the maximum is along the crystallographic b-axis, (see references [2, 3]) as shown in Figure S4a. Due to the similar spot size and strip widths, to properly determine the WTe_2 crystal axes, the $CrPS_4$ background was subtracted for each measurement. Furthermore, the intensity ratio from peaks P6 and P7, at ∼165.7cm^{-1} and ∼211.3 cm^{-1} respectively, determines the crystallographic a-axis of WTe_2 [1, 4–6]. From fitting our data to a $\cos^2(\alpha)$ function, we determine that the strips are aligned at $\alpha \sim -12.08°$ from the crystallographic a-axis of WTe_2.

II. EXCLUDING PARASITIC CONTRIBUTIONS TO THE NON-LOCAL RESISTANCE

II.A. Excluding Magnetoconductance of $CrPS_4$

The low temperature electronic transport properties of $CrPS_4$ have been investigated in which a magnetoconductance of upto 5000 % is observed for certain applied gate voltages and an applied external field of 10 T [7]. If the observed non-local resistance modulation was due to the magneto-transport properties of $CrPS_4$ arising from a parasitic leakage charge current, then the expected non-local resistance modulation observed is expected to go as $R_{NL} \propto \alpha(1 + \beta B^2)$, which is in contrast to the observed field dependence of R_{NL}, which has a sharp increase in the non-local resistance corresponding to the spin-flip transition of $CrPS_4$. Thus, we conclude that the observed non-local resistance modulation cannot arise due to the magneto-transport properties of $CrPS_4$. Additionally, a non-local resistance modulation arising due to a parasitic charge current in the two-dimensional magnet should also result in a non-local resistance modulation when the injector and detector are both platinum (assuming the same order of magnitude of contact resistances) for an out-of-plane field sweep (since $CrPS_4$ shows a B^2 magnetoconductance for a magnetic field applied out-of-plane), which was not observed in the earlier reports [8, 9].

II.B. Excluding Parasitic Leakage Current

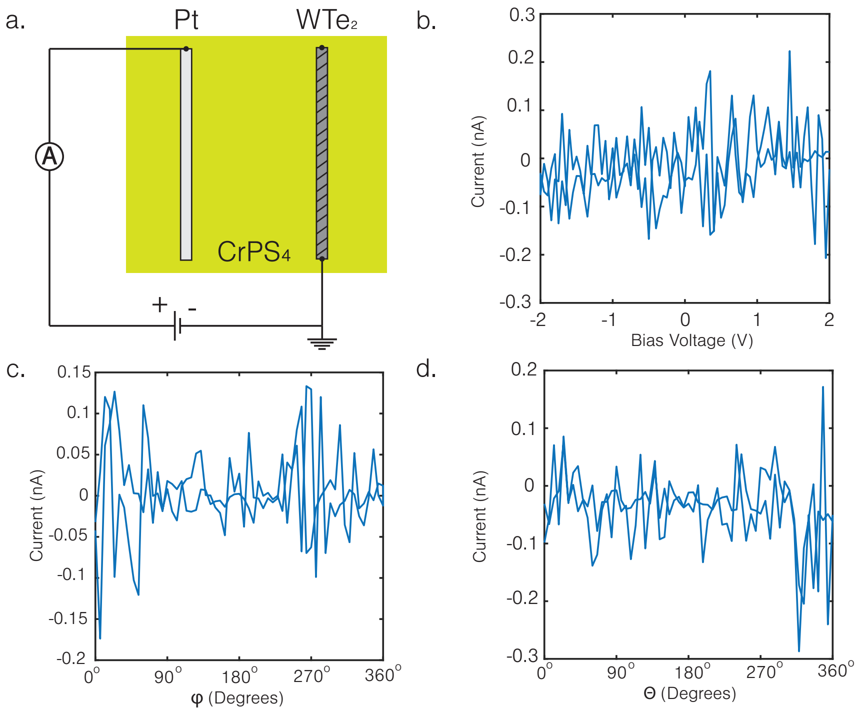

FIG. S5: a) The equivalent measurement circuit for electrical short check across the strips, b) the measured IV with an applied external magnetic field of +7.75 T with $\varphi = 0$; the current measured with a 2V D.C. bias across the strips for c) out-of-plane, d) in-plane angular rotation with an applied field of +7.75 T.

To exclude any contribution from parasitic leakage current and additionally ensure that $CrPS_4$ is insulating between the Pt and the WTe_2 strip, additional DC measurements were performed. A Keithley 2400 (input impedance $> 10\text{G}\Omega$) was used as a voltage source to apply a 2V bias across the contacts as shown in in Fig. S5a. The VI characteristics recorded across the strip is shown in Fig. S5b. We observe no detectable current up to 2V indicating the insulating behavior across the strips and thus conclude that WTe_2 is fully ethced. Additionally, with the application of 2V across the strips and an applied field of 7.75 T, angular dependent measurements were performed for both in-plane and out-of-plane angular rotations as shown in Fig. S5c,d. We observe no angular dependence of the current and thus conclude that the non-local resistance modulation cannot be due to magnetoresistance effects

of $CrPS_4$ arising due to a parasitic charge current leakage between the strips.

Apart from checking for electrical shorting by applying a DC voltage across the strips, further verification was performed by means of inverting the grounding configuration across the voltage detector strip. The grounding convention used for the measurements of the non-local voltage voltage is depicted by means of a simplified circuit as shown in Fig. S6a. In the presence of any parasitic charge leakage across the strips, the current leaks to the ground on the voltage detector, which for the conventional grounding, flows through the resistive element of the circuit (where the insulating $CrPS_4$ is simplified as R_C). Upon switching the voltage detector's grounding, the parasitic leakage charge current now additionally flows through the WTe_2 strip as illustrated in Fig. S6b (the elements encountered by the leakage current are highlighted in red). While performing a field scan, this additional WTe_2 resistance in the leakage path, owing to its magnetoresistance, would give rise to a different response. The non-local voltages for both grounding configurations while injecting with Pt at 25K are shown in Fig. S6c.

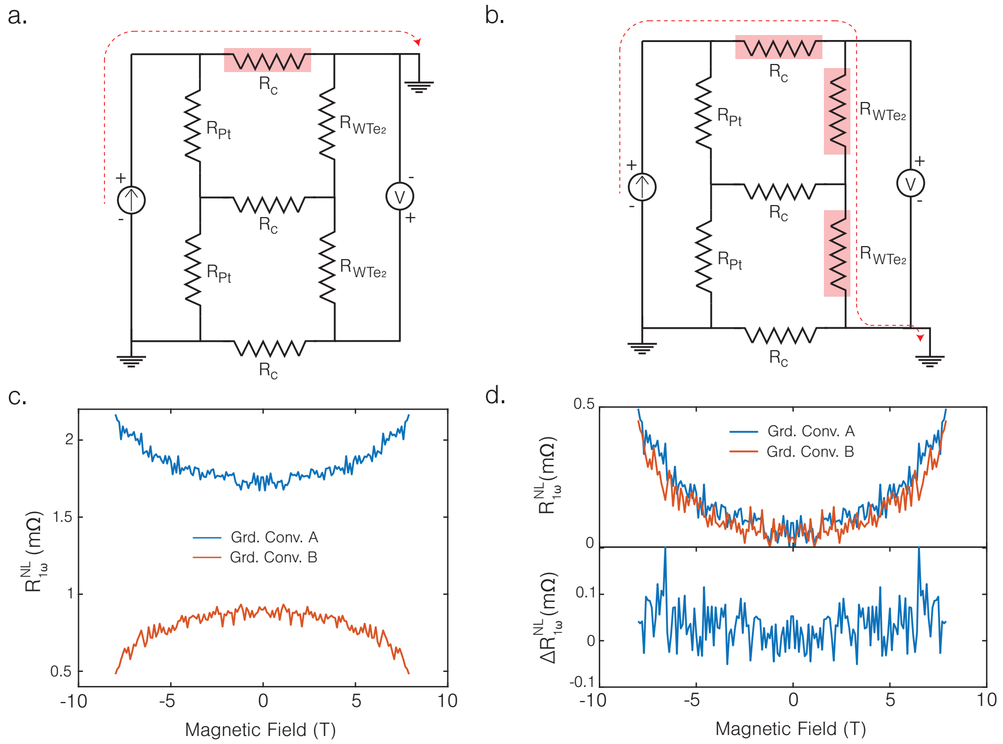

FIG. S6: The simplified equivalent circuit for a) the non-local measurement geometry with the grounding configuration for the measurement of the non-local voltage, b) with the ground configuration swapped, c) the field dependence (at $\varphi = 45^o$) of the non-local voltage, d) the signals superimposed onto each other and their difference ($i_{bias} = 130$ μA, Temp = 25K).

It is observed that there is an offset on the measured signal that is not dependent on the grounding of the voltage detector and an offset that is grounding dependent. Furthermore, the measured signals switch signs and additionally for the grounding convention of Fig. S6b. The parasitic leakage current through WTe_2 combined with the magnetoresistive properties of WTe_2 would give rise to a positive B^n dependence which we do not observe. The measured signals corrected for the offset and grounding convention, superimposed on each other are shown in Fig. S6d. We thus conclude that the measured non-local voltage cannot be due to a parasitic leakage charge current. The effect of capacitive coupling across the strips in combination with the effect of the MR of WTe_2 is discussed in detail in Section V.

III. EXPECTED NON-LOCAL RESPONSE DUE TO MAGNON TRANSPORT

In platinum, a charge current along the strip is converted to a spin accumulation polarization parallel to the interface (at the Pt/$CrPS_4$) interface, where the charge-to-spin interconversion arises from the conventional spin hall effect. Any additional mechanism in the charge-to-spin (or vice versa) interconversion would result in additional components of polarization of the spin accumulation at the interface. With WTe_2 possessing a rich spin texture [10], the underlying spin-to-charge interconversion mechanism is expected to play a vital role in the detection of any non-local resistance modulation due to magnon transport. When a charge current flows through the injector (Pt), the charge-to-spin interconversion process (SHE) produces a transverse spin current, leading to a net spin accumulation at the injector/magnet interface with the polarization determined by the charge-to-spin interconversion process. Depending on whether the polarization of this spin accumulation is parallel or antiparallel to the magnetization **M**, magnons are either annihilated or excited, respectively, resulting in a non-equilibrium magnon density in the magnet [11]. This imbalance drives magnon diffusion through the magnet, thereby transporting spin angular momentum. At the detector, the reciprocal process takes place: magnons interact at the interface, flip electronic spins, and induce a spin accumulation in the detector, which is then converted into a charge current owing to the spin-to-charge interconversion mechanism of the detector, which under open-circuit conditions, manifests as a measurable voltage (V). This is schematically illustrated in Fig. S7a. We emphasize that this remains valid for arbitrary polarizations of the spin accumulation and magnetization direction, with the effective excitation and detection determined by the projection of the polarization of the spin accumulation onto the magnetization direction. We expect any additional charge-to-spin interconversion mechanisms (apart from the conventional SHE) to be reflected in the relative phases between the local second harmonic response (local Spin Seebeck Effect (local SSE) across Pt) and the non-local first harmonic response (in the angular rotations of the magnetic field).

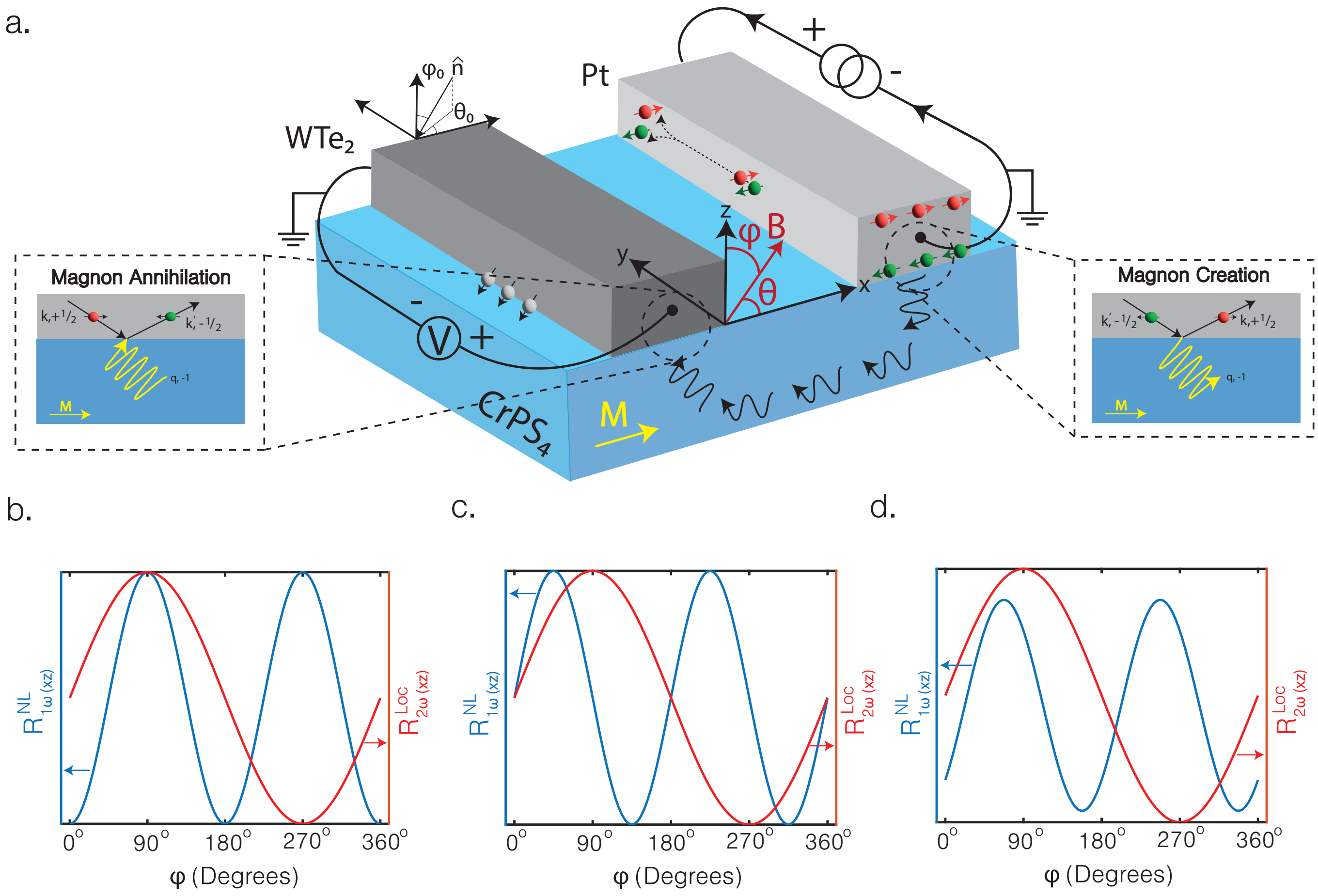

FIG. S7: a) Illustration of injection of magnon spins by Pt via conventional spin hall effect and the detection of magnon spin by WTe_2 sensitive along the direction $\hat{n}$. Excepted second harmonic response of plantinum (local SSE) and the corresponding non-local first harmonic response for $\hat{n}$ along b) (100), c) (010) and d) (110) for out-of-plane angular rotations.

The expected non-local first harmonic response across the WTe_2 strip due to magnon transport excited by a Pt strip is shown in Fig. S7. In WTe_2, the spins polarized along $\hat{n}$ are transformed into an open-circuit voltage (due to the spin-to-charge interconversion) and thus $\hat{n}$ defines the effective axis for the spin-to-charge conversion process and Θ_0, φ_0 are the corresponding angles of $\hat{n}$ in spherical coordinates. For only conventional SHE, $\hat{n} = \hat{x}$ ($\Theta_0 = 0$, $\varphi_0 = 90$). Here, it is assumed that Pt can only inject spins polarized along $\hat{x}$ and that the sign of the spin hall angles of Pt and WTe_2 are both positive. For the spin texture of WTe_2 sensitive to magnon spin current polarized in arbitrary directions, it can be noted that the first harmonic non-local response obtains a phase shift with respect to the local second harmonic response across Pt for out-of-plane angular rotations of the magnetic field. This can be seen as follows:

$$\Delta R_{NL} \propto (\vec{M} \cdot \hat{x})(\vec{M} \cdot \hat{n}), \tag{1}$$

where the first term corresponds to the projection of the polarization of the spin accumulation of Pt onto the magnetization ($\vec{M}$) of $CrPS_4$ and the latter the contribution from WTe_2. Analogous to the argument presented above, any component of polarization of the spin accumulation along the direction of the strip will result in a phase shift for in-plane angular rotations (in the xy plane).

Furthermore, comparison of the local signal (spin hall magnetoresistance (SMR) or the local Spin Seebeck Effect (local SSE)) with the detected non-local voltage provides information on the sign of the spin hall angle. Fig. S8 depicts the expected SMR and the non-local voltage modulation for different signs of the spin hall angles.

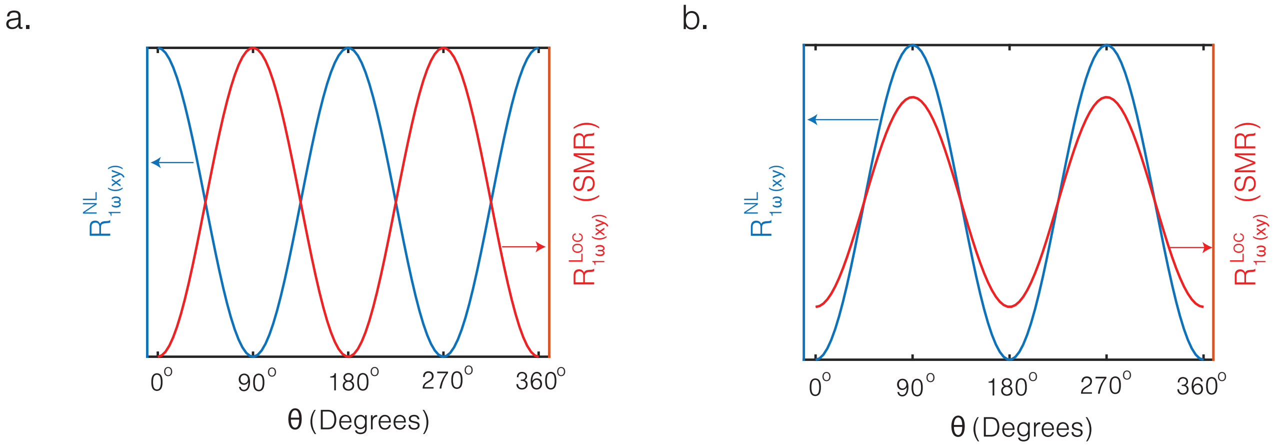

FIG. S8: Illustartion of the expected spin hall magnetoresistance and the non-local voltage as a function of in-plane angular rotations for a) same sign and b) opposite sign of the spin hall angles.

This can be seen from the dependency of the detected signals on the spin hall angles, namely,

$$\Delta R_{Loc}(SMR) \propto [\Theta_{Pt}]^2, \tag{2}$$

$$R_{NL} \propto \Theta_{inj}.\Theta_{det} \propto \Theta_{Pt}.\Theta_{WTe_2}. \tag{3}$$

Similar analogy of extracting the sign of the spin hall angle of the detector can be extended to the local SSE where $\Delta R_{2\omega} \propto \Theta_{Pt}$. Thus, from the signs of the local and the non-local responses we can further determine the sign of the spin hall angle of WTe_2.

IV. DATA PROCESSING AND FITTING PROCEDURE

The interquartile range (IQR) is a statistical measure of spread that represents the range within which the central 50% of a dataset lie, and is defined as the difference between the third quartile (Q_3) and the first quartile (Q_1), *i.e.*, IQR = Q_3 - Q_1. To identify outliers, the measured data were first fit to the expected angular dependence (π or 2π periodic functions), and the residue were analyzed. Data points with residuals below (Q_1 - 1.5×IQR) or above (Q_3 + 1.5×IQR) were considered outliers and excluded from further analysis, where Q_1 and Q_3 correspond to the first and third quartiles of the residual distribution, respectively.

IV.A. Symmetrization and Anti-Symmetrization of Data

For the fitting of angular rotations, the obtained data were symmetrized for the first-harmonic responses and anti-symmetrized for second-harmonic responses with the measurements of opposite fields. The symmetrization and anti-symmetrization of the data was performed by:

$$\begin{aligned} R_{sym} &= \frac{R(+B) + R(-B)}{2}, \\ R_{anti-sym} &= \frac{R(+B) - R(-B)}{2}. \end{aligned} \tag{4}$$

The presence of any DC offset in the applied current source results in a mixing of the second order response into the first order response. For instance, the non-local voltage measured across the WTe_2 strip for the out-of-plane angular rotations, the detected voltage suffers from the leakage of a large second order response (attributed to Nernst effect) into the first order response. The field dependencies of the first and second harmonic responses being different in the angular rotations, namely being π periodic and 2π periodic respectively, enable us to decouple the two. All the plots obtained are symmetrized for first harmonic responses and anti-symmetrized for second harmonic responses unless mentioned otherwise.

IV.B. Fitting Procedure and Error bar estimation

The fitting processes involved optimizing three parameters, namely the amplitude, angular phase shift, and the offset which were optimized within specific bounds. The amplitude was restricted to be positive, *i.e.* [0, ∞), the phase was free for second harmonic responses and [0,180] for first harmonic responses, and the offset was allowed to vary freely. The error bars on the amplitude obtained from the fitting of the data used is the standard error of the fitted amplitudes, which was estimated using the 95% confidence interval (obtained from nonlinear least squares fitting). The standard errors (σ) were then approximated from the width of the confidence interval assuming a normal distribution (of the residue, *i.e.* assuming a Gaussian noise).

$$\sigma = \frac{\text{Confidence Interval Width}}{3.92}. \tag{5}$$

The length of the total error bar in the graphs correspond to a length of 2σ, where σ is defined in Eqn. 5.

IV.C. Removal of Rotator Offset

For in-plane angular rotations, a 29° offset between the sample (mounted on the sample rotator) and the magnetic field was determined from the local SSE measurements across Pt and was corrected for in the angular rotation data. For out-of-plane angular rotations, no offset was corrected in the rotation angle.

V. ANALYSIS OF OUT-OF-PLANE FIELD ROTATIONS

V.A. Magnetoresistance of WTe_2

The two-terminal magnetoresistance (MR) of WTe_2 is shown in Fig. S9. The MR as a function of the applied field for varying temperature (external field along the c-axis) is shown in Fig. S9a and for varying angle of the applied field is shown in Fig. S9b. The modulation of the two-point resistance was observed to not be quadratic as a function of the applied field and was fit with the function:

$$\Delta R = A.B^n, \tag{6}$$

where B is the strength of the applied magnetic field, A the corresponding scaling coefficient and the exponent n. The dependency of A and n are plotted in Fig. S9c,d as a function of temperature and the magnetic field angle respectively. The 2-point resistance (in the absence of a magnetic field) of WTe_2 measured at 15K, 25K, 40K, 50K, 60K and 80K were 0.686 kΩ, 0.838 kΩ , 1.13 kΩ , 1.34 kΩ , 1.72 kΩ and 2.3 kΩ respectively.

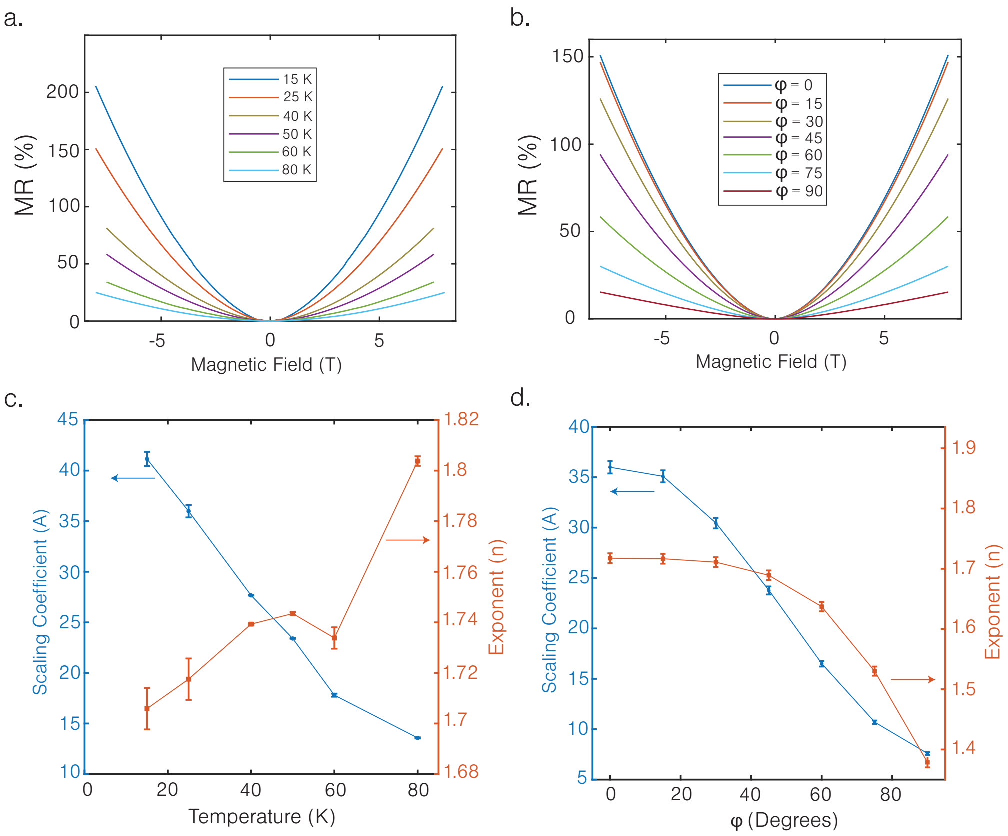

FIG. S9: The magnetoresistance measured across WTe_2 as a function of the applied magnetic field for varying a) temperature, b) out-of-plane angle at 25K; the dependence of the fit amplitude for the pre-scaling factor and the exponent as a function of c) temperature, d) the out-of-plane magnetic field angle (i_{WTe_2} = 50 μA).

A similar MR behavior for WTe_2 as a function of the temperature and angle of the applied field has been reported in Ref. [12], which has been attributed to WTe_2 being electronically 3D and exhibiting a mass anisotropy for an applied field parallel and perpendicular to the c-axis. Furthermore, the temperature dependence of the MR has been attributed to the Fermi liquid state [12].

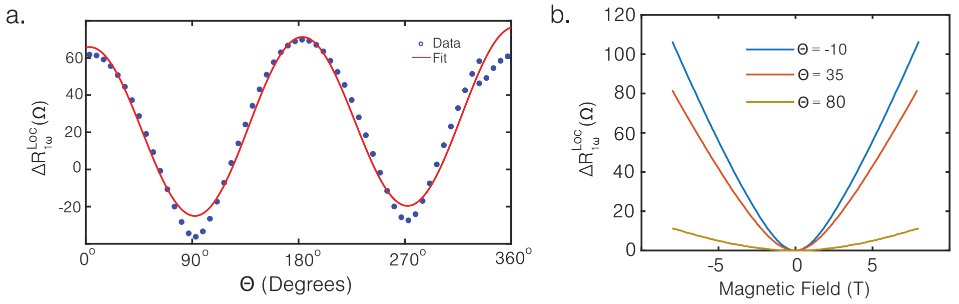

FIG. S10: a) The angular dependence of the local 2-point resistance of WTe_2 as a function of the applied in-plane magnetic field at 25K and 8T and b) the field dependence of the in-plane MR for various in-plane field directions.

Similarly, for a magnetic field applied in-plane, an in-plane MR was observed as shown in Fig. S10. The maximum resistance in the angular rotations is observed when the applied magnetic field is perpendicular to the current in the strip. We note that measuring spin-hall magnetoresistance (SMR) while injecting with WTe_2 is not possible owing to the large MR of WTe_2. SMR typically of the order of 0.3 Ω (for platinum) is overshadowed by the MR of WTe_2 and extraction of the information about spin injection from local measurements is not possible.

V.B. Effect of MR of WTe_2 on the non-local voltage

In the non-local geometry, for an ac current across the platinum strip, an illustration of the geometry and a simplified circuit are shown in Fig. S11a,b. Due to an ac current across the platinum strip, through the capacitive coupling (attributed to $CrPS_4$ and the capacitive coupling across the strips), for a finite impedance of the capacitive elements, a current flows through the non-local WTe_2 strip, where due to the large MR of the material, a modulation in the out-of-phase (Y-) component is observed. In Fig. S11c,d the non-local resistance modulation of the out-of-phase component is shown as a function of angular rotation in the out-of-plane direction and a magnetic field scan respectively.

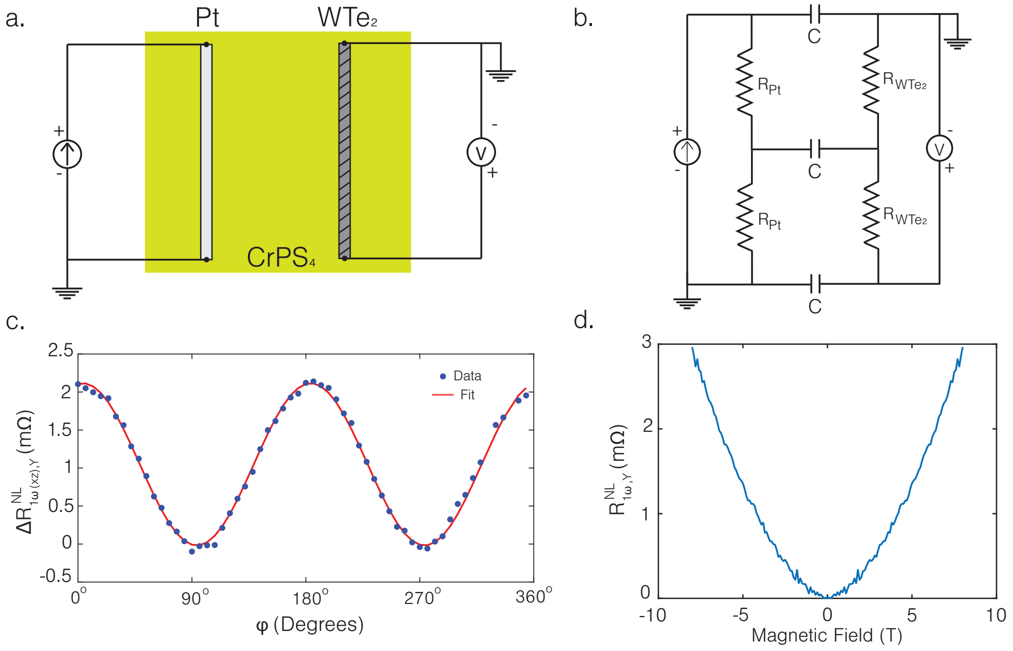

FIG. S11: a) Illustration of the non-local measurement geometry and b) a simplified equivalent circuit for the same; the out-of-phase component of the non-local resistance as a function of c) the out-of-plane angle (at 7T applied field) and d) the applied field (frequency of 3.33 Hz, at 25K).

The observed field dependence of this out-of-phase component follows a similar relation to that in Eqn. 6, with

n=1.69±0.01, similar to the field dependence of MR across WTe_2 at 25K (with n=1.716±0.001). At 25K and an applied external field of 7T, the modulation of the out-of-phase component is about 2.1 mΩ. Solving for the capacitance, using the 2-point resistances of platinum as 2.8 kΩ and WTe_2 as 838 Ω and taking into account the MR of WTe_2 at 7T and 25K, we estimate the capacitance as C $\simeq$ 25 pF.

Compared to the out-of-plane angular rotations where the difference in MR is about 895 Ω, the difference in the MR for in-plane angular rotations is about 80 Ω at 8T, which results in a significantly smaller modulation in the Y-component for in-plane angular rotations compared to the out-of-plane angular rotations of the magnetic field.

V.C. Decoupling parasitic effect of the MR from the detected non-local voltage

As discussed in the previous section, the MR of WTe_2 combined with the capacitive coupling leads to a modulation of the out-of-phase (Y-) component of the voltage measured across WTe_2. With any instrumental cross-talk that leads to a parasitic leakage of the Y-component into the in-phase component (X-) component, causes a mixing of the contribution from MR to the non-local voltage that could contain information from the magnonic transport in the device. To understand the nature of the cross-talk, frequency dependent angular rotation of the magnetic field were performed at 25K (where the driving frequency of the current in the Pt strip ($i_0 \sin(\omega t)$) was changed). The fit amplitudes obtained for the Y and X components are shown in Fig. S12.

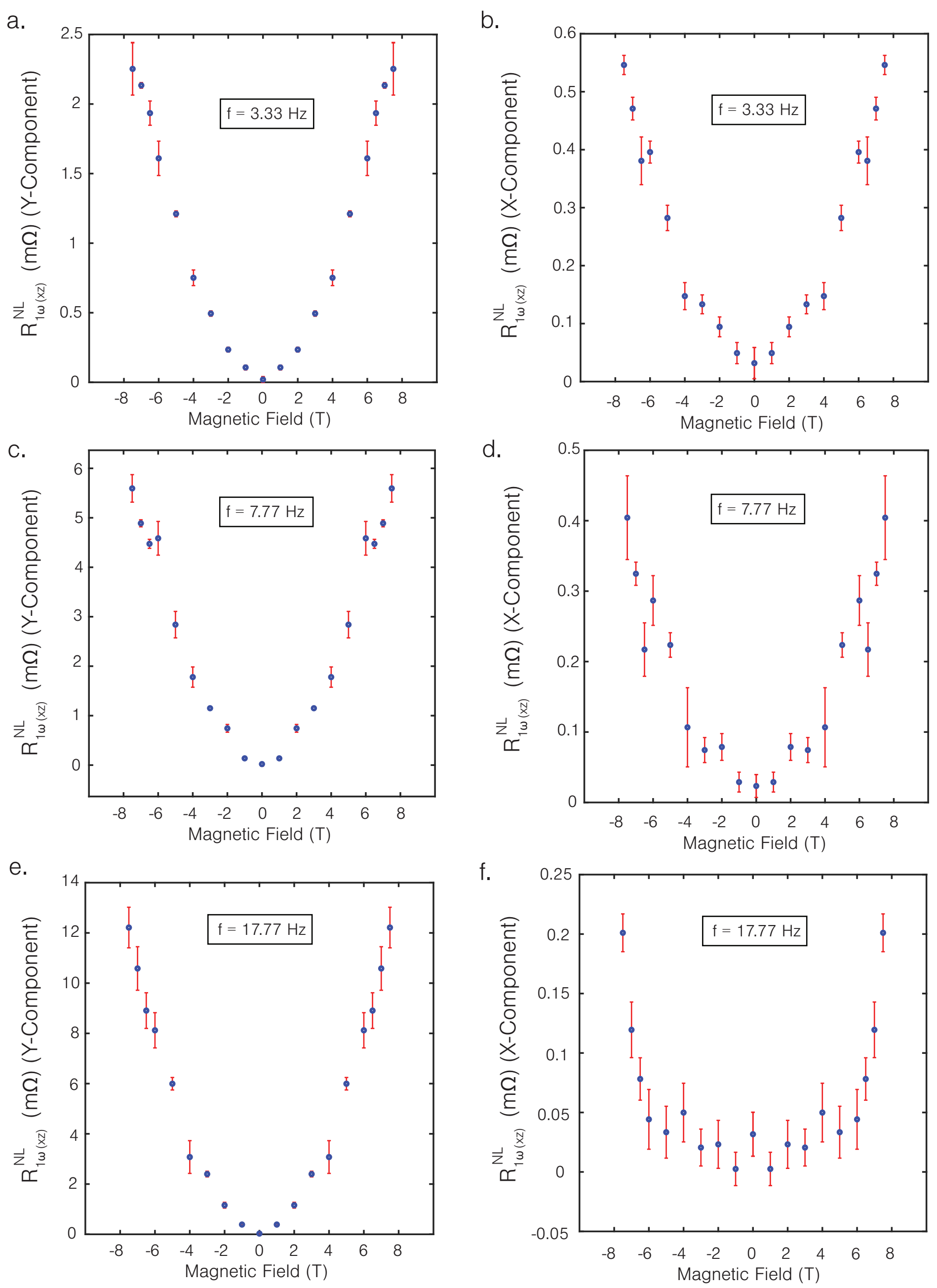

FIG. S12: For an out-of-plane angular scan at 25K, the field dependence of the amplitude of modulation for the a,c,e) out-of-phase component at 3.33 Hz, 7.77 Hz and 17.77 Hz respectively and b,d,f) in-phase component at 3.33 Hz, 7.77 Hz and 17.77 Hz respectively (i_{Pt} = 100 μA).

We note the offset on the signal measured for the Y-component as 2.3 mΩ, 5.9 mΩ and 12.6 mΩ for a frequency of 3.33 Hz, 7.77 Hz and at 17.77 Hz respectively.

The cross-talk between the X- and the Y- components can be expressed as:

$$V_{X,Measured} = V_X + \epsilon V_Y, \tag{7}$$

$$V_{Y,Measured} = V_Y + \eta V_X, \tag{8}$$

where ϵ and η characterize the cross-talk between the X and the Y components of the measured voltage. The angular dependency of V_Y for out-of-plane angular rotations of the field (attributed to the combined effect of the capacitive coupling between the strips and the MR of WTe_2) is given as:

$$V_Y(\varphi) = V_0^Y + V_1^Y \cos^2(\varphi - \frac{\pi}{2}). \tag{9}$$

The leakage of Y-component into X would thus have the same angular dependency as $V_Y(\varphi)$ and can be removed from $V_{X,Measured}$. This is done by fitting $R_{Y,Measured}(\varphi)$ first and then fiting $R_{X,Measured}$ with a fixed phase as the one obtained from $R_{Y,Measured}(\varphi)$ followed by a free fit of the residue. This is effectively:

$$\begin{aligned} R_{Y,Measured}(\varphi) &= R_0^Y + R_1^Y \cos^2(\varphi - \varphi_0), \\ R_{X,Measured}(\varphi) &= [R_0^X + R_1^{Y,X} \cos^2(\varphi - \varphi_0)] + R_{NL}^X \cos^2(\varphi - \varphi_0 - \frac{\pi}{4}). \end{aligned} \tag{10}$$

We note that this form of fitting the residue after the removal of a fixed phase (obtained from the Y-component) always results in a π periodic function phase shift by $\pi/4$. This can be seen as follows:

$$\begin{aligned} f(\varphi) &= a + b\cos^2(\varphi + \beta), \\ &= a + \frac{b}{2}(1 - \cos 2\beta + \sin 2\beta) + b\cos 2\beta \cos^2\varphi - b\sin 2\beta \cos^2(\varphi - \frac{\pi}{4}). \end{aligned} \tag{11}$$

This can also be seen through basic Fourier analysis, where a π periodic function is decomposed into a linear combination of $\sin 2x$ and $\cos 2x$ (and their higher harmonics). Physically, the contribution of in-plane polarized magnon spin injection and in-plane polarized magnon spin detection would result in a $sin^2\varphi$ (*i.e.* 1-$\cos^2\varphi$) contribution in the out-of-plane angular rotation, which is lost in removing the instrumental cross talk. Alternatively, the injection of in-plane polarized magnon spins and detection of out-of-plane polarized magnon spins (or vice-versa) would result in a $\pi/4$ phase-shifted signal ($\sin 2\varphi \sim \cos^2(\varphi - 45)$).

The implementation of this fit procedure to extract R_{NL}^X (with Pt as the injector and WTe_2 as the non-local detector) is shown in Fig. S13 for angular rotations at 15K. Fig. S13a,b show the modulation of the Y-component in the angular scans and their corresponding fit for different applied fields of 8T and 6T. At 8T the magnetic sublattices of $CrPS_4$ are fully saturated and collinear compared to that at 6T. The fit with this fixed phase on the measured R_{NL}^X is shown in Fig. S13c,d. It can be seen that the measured in-phase non-local resistance at lower applied external magnetic fields is predominantly due to the parasitic leakage of the Y-component into X. However, for higher fields, namely, past the spin-flip field, an additional 45 degree phase shifted signal is observed as shown in Fig. S13 e,f.

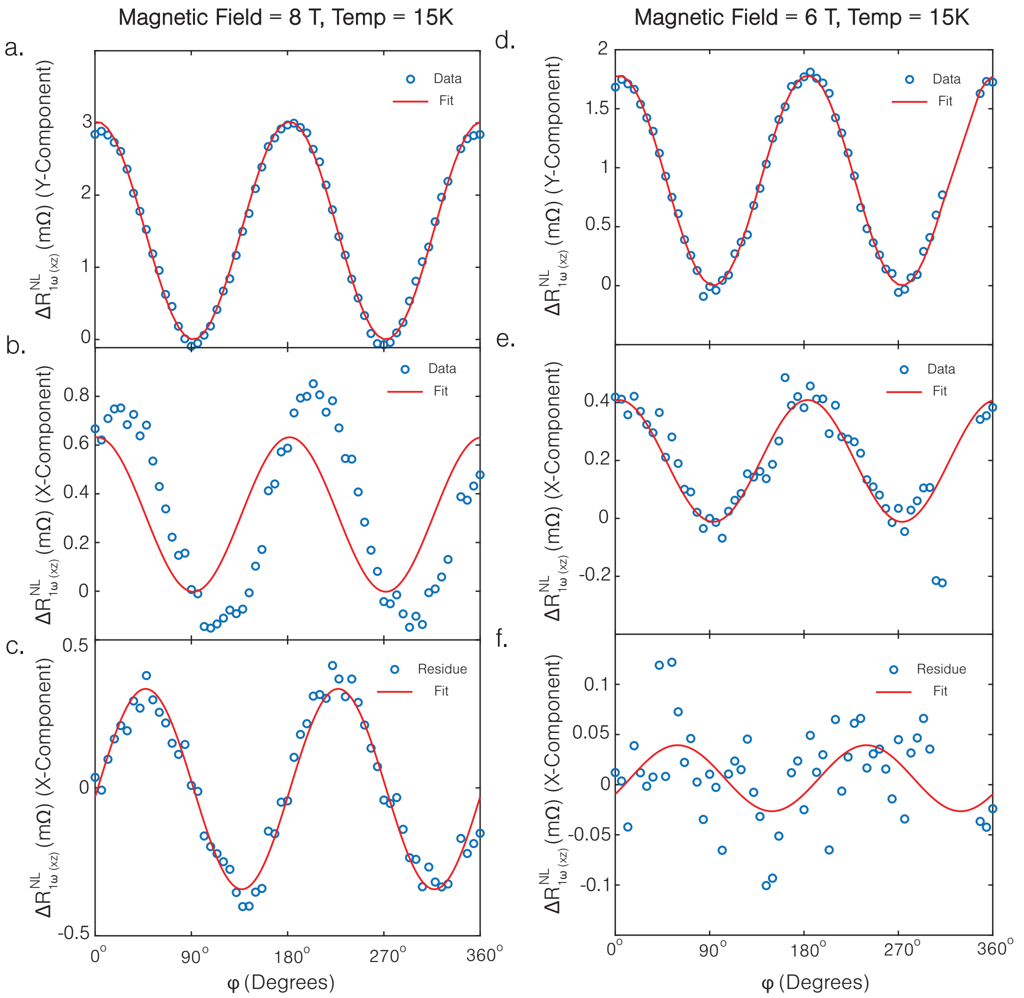

FIG. S13: For an out-of-plane angular rotations at 15K and applied fields of 8T and 6T and a,b) the modulation in the Y-component and its corresponding fit, c,d) the X-component and a fit with the phase fixed to those obtained from a,b respectively, e,f) the residue from c,d and its fit ($i_{Pt} = 100\ \mu A$).

The amplitude of the modulation of the Y-component obtained is shown in Fig. S14 a. The amplitude of the modulation of the X-component obtained in the absence of the removal of $R_1^{Y,X}$ is shown in Fig. S14b (*i.e.* a free fit) while the field dependence of $R_1^{Y,X}$ and R_{NL}^X are shown in Fig. S14 c,d, where in Fig. S14 d we observe a rapid onset of this additional contribution as a function of the applied magnetic field corresponding to the spin flip field of $CrPS_4$. The above mentioned fit procedure is used to obtain the values of ΔR_{NL} used in the main text for various temperatures and applied fields.

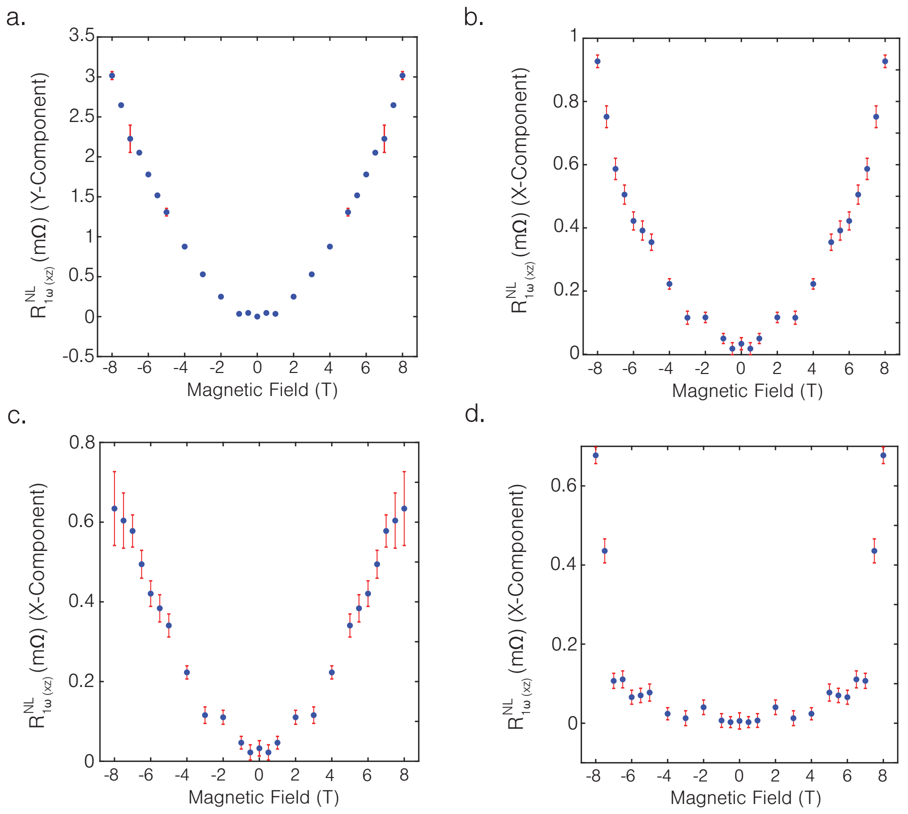

FIG. S14: The field dependence of the amplitude of the non-local response modulation across WTe_2 of a) the Y-component, b) R_{NL} with a free fit, c) the amplitude of modulation with the same phase as R_1^Y and d) the amplitude of the residue (i_{Pt} = 100 μA, 15K).

VI. VERIFICATION OF LINEAR RESPONSE AND RECIPROCITY

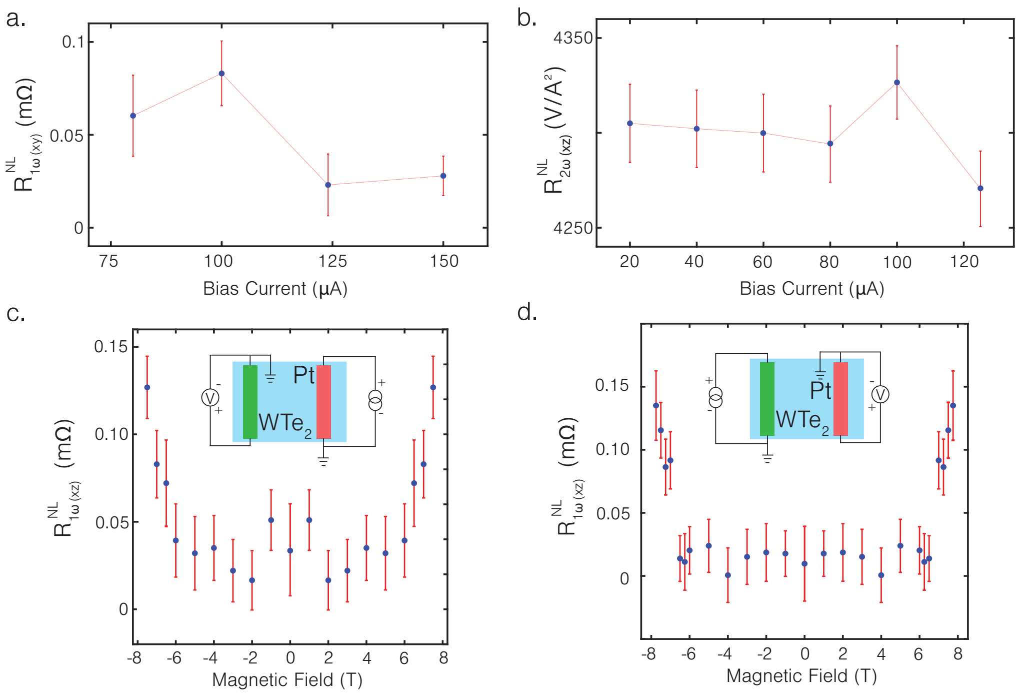

FIG. S15: The bias dependence of the amplitude of the non-local response across WTe_2 for a) the first harmonic (in-plane angular rotation), b) second harmonic (out-of-plane angular rotation) at 25K and an applied field of 8T; the field dependence of the non-local first harmonic response for the out-of-plane angular rotations while injecting with c) Pt and d) WTe_2 at 25 K (i_{Pt/WTe_2} = 100 μA).

The amplitude of the first harmonic non-local resistance modulation for in-plane angular rotations and the amplitude of the non-local second harmonic response for out-of-plane angular rotations are shown in Fig. S15a,b respectively. Based on the bias dependence, we conclude that up till 100 μA of bias current, the response falls within the linear response regime of the system.

The amplitude of the first harmonic non-local resistance modulation for out-of-plane angular rotations are shown in Fig. S15c,d for injection with Pt strip and detection with WTe_2 strip and vice-versa respectively. The value of the amplitude of the non-local first harmonic modulation while injecting with platinum and WTe_2 ($i_{bias} = 100\mu A$) at 25K are 0.126 ± 0.018 and 0.115 ± 0.022 mΩ respectively at 7.5T which is consistent with reciprocity relations.

Having checked for reciprocity, the amplitude of non-local first harmonic response in the out-of-plane angular rotation, we use the reciprocal measurement for 8T at 25K (in Fig. 5 of the main text), *i.e.* use the amplitude for injecting with WTe_2, whereas the other data points are while injecting with the Pt strip.

VII. SPIN HALL MAGNETORESISTANCE (SMR) OF PLATINUM

The local first and second harmonic responses measured across Pt for in-plane and out-of-plane angular rotations of the magnetic field are shown in Fig. S16a, b. The first and second harmonic responses are measured simultaneously. We observe that the sign of SMR while consistent and as expected for Pt for in-plane angular rotations (and consistent with the corresponding local SSE) has a negative sign for the out-of-plane angular rotation.

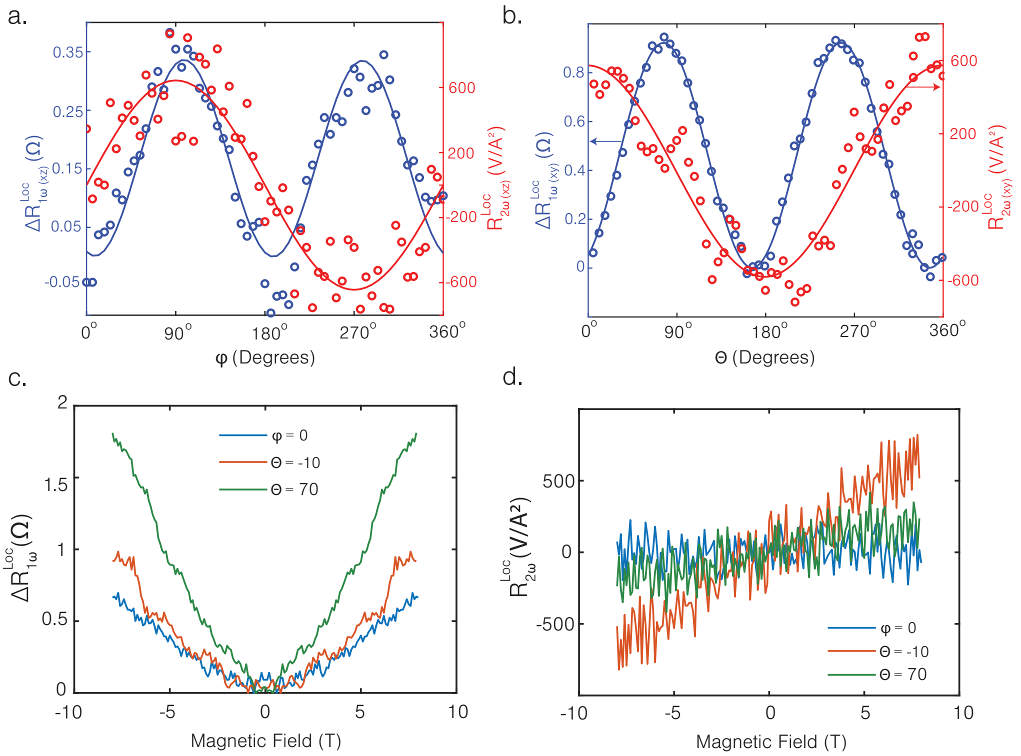

FIG. S16: The measured SMR and local SSE across Pt for a) out-of-plane angular rotations, b) in-plane angular rotations for an applied field of 8T and the field dependence of c) SMR and d) local SSE at 25K (i_{Pt} = 100 μA).

It is puzzling that for out-of-plane angular rotations the corresponding first harmonic response (interpreted as SMR) and the local second harmonic response (interpreted as local SSE) seem to be incompatible with each other. To further understand this, field scans of the first and second harmonic responses were performed along different applied field directions as shown in Fig. S16c,d. We note that for Θ = 70, almost along the strip, the measured first harmonic response is approximately the sum of the responses measured for φ = 0 (perpendicular to the strip out-of-plane) and Θ = -10 (almost perpendicular to the strip in-plane).

VIII. ANALYSIS OF SECOND HARMONIC RESPONSES

VIII.A. Local Second Harmonic response of WTe_2

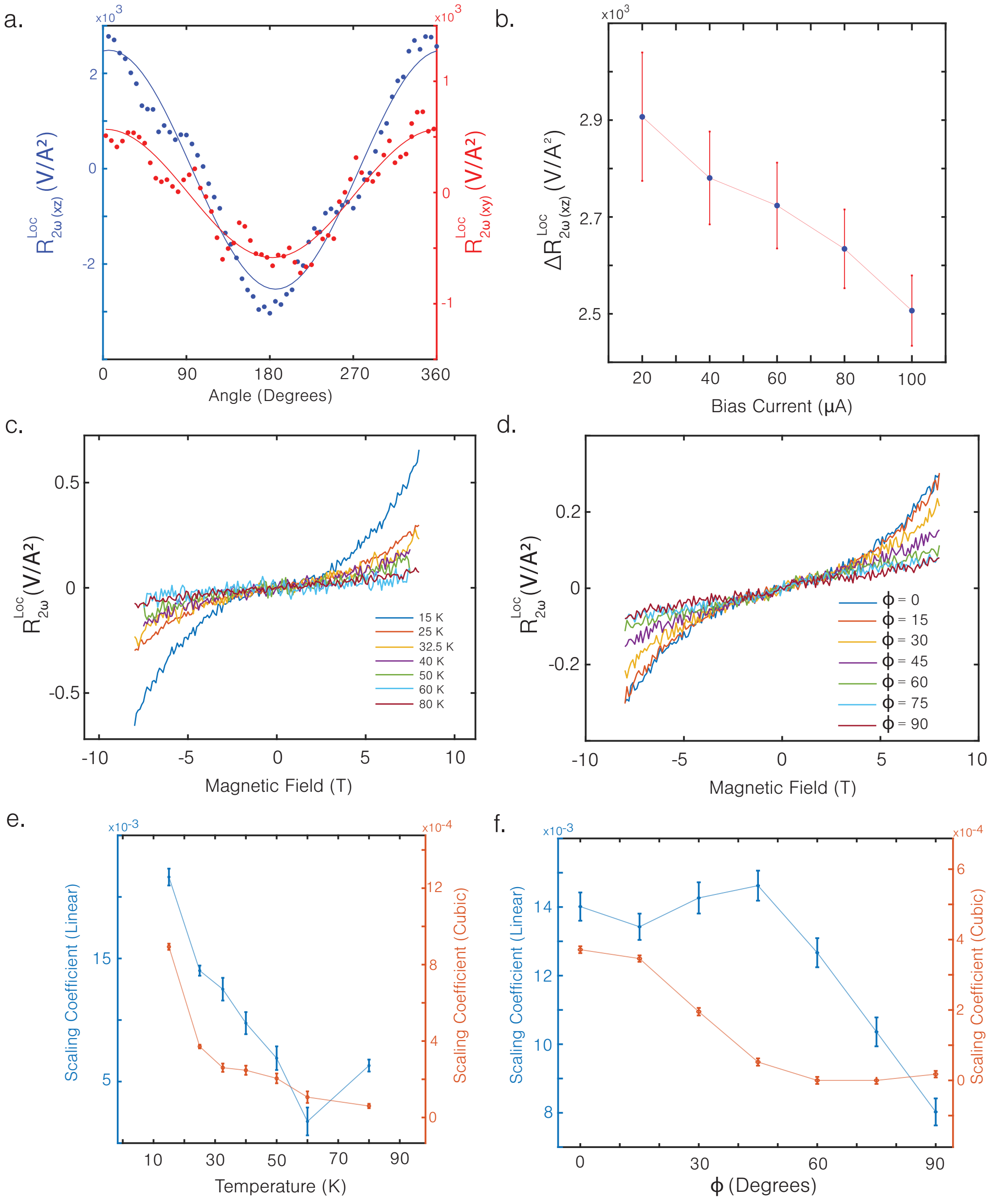

FIG. S17: a) Local second harmonic response of WTe_2 as a function of in- and out-of-plane angular rotations (B = 7.75 T), b) the bias dependence of the local second harmonic across WTe_2 for out-of-plane angular rotations (B = 7.75 T); the field dependence of the local second harmonic resistance for various c) temperature, d) varying out of plane magnetic field angles; the fit amplitudes corresponding to the linear and cubic dependence in magnetic field as a function of e) temperature, f) the out of plane magnetic field angle (at 25K, i_{WTe_2} = 100 μA).

The local second harmonic response measured across WTe_2 is shown in Fig. S17. We note that the angular dependency of $R_{2\omega}$ for in-plane magnetic field originates from bilinear magnetoresistance [1], which is intrinsic to WTe_2 and that this overshadows the local SSE present (if any).

We observe that the measured local second-harmonic response deviates from a linear behavior for higher magnetic

fields and lower temperatures. We attribute this to the thermal gradient from Joule heating of the WTe_2 being field dependent. This can be seen as:

$$\nabla T \propto i^2 R \propto i^2[R_0 + A.B^n cos^2(\varphi)], \tag{12}$$

where the applied magnetic field changes the two-point resistance of WTe_2 and thus the thermal gradient. The second harmonic response was thus fit with:

$$R_{2\omega} = A_1 B + A_3 B^3, \tag{13}$$

where A_i corresponds to the scaling coefficient. The temperature and magnetic field angle dependency of A_i are shown in Fig. S17 e, f. We note that the additional cubic dependency follows a similar trend to that of the MR across WTe_2.

VIII.B. Non-Local Second Harmonic response of WTe_2

While injecting with Pt, the measured non-local second harmonic response is shown in Fig. S18a,c for varying angles of the applied field. For an out-of-plane applied field, the second-harmonic response is dominated by the Nernst effect, which has been reported for WTe_2 [13] and more recently for a wider range of Te-based van der Waals materials [14]. To verify if there are additional effects overshadowed by the Nernst effect (such as non-local spin seebeck effect), we subtract a linear background, by fitting the data acquired at high magnetic fields (B $\in$ [6.5T, 8T]), since any magnetization dependent response would be saturated at these high fields and the response would arise from Nernst effect alone. The residue resulting after removal of this linear fit is shown in Fig. S18b.

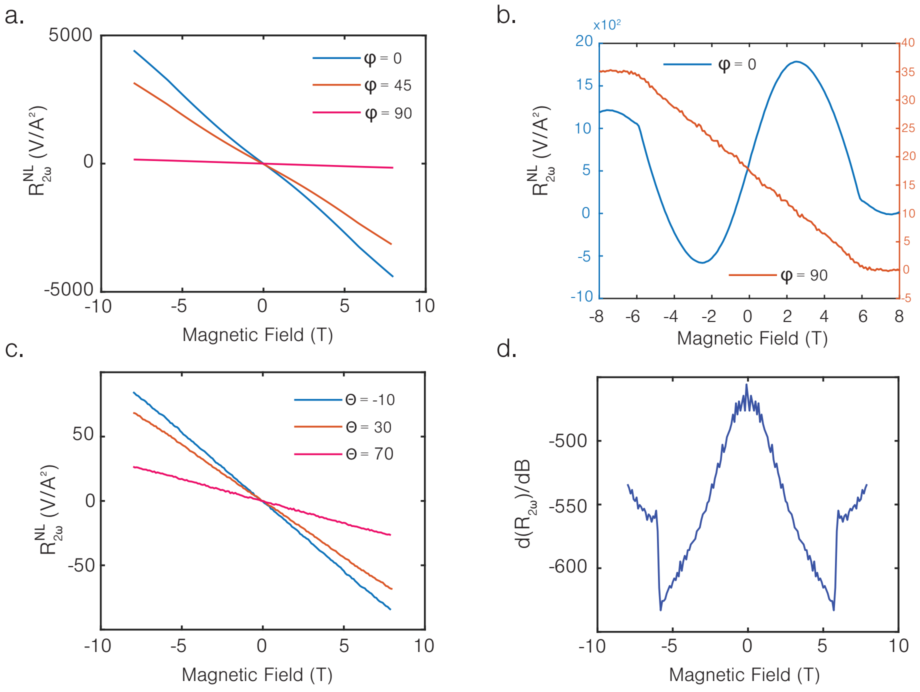

FIG. S18: The field dependence of the measured non-local second harmonic across WTe_2 for a) various out-of-plane angles, c) various in-plane angles; b) the second harmonic response after a linear background removal, d) the derivative of the second harmonic response as a function of field for $\varphi = 0$ (at 25K and $i_{Pt} = 100\ \mu$A).

We note that the form of the residue depends on the region that is fit to obtain the slope of the linear background (fitting of the low-field data to remove the linear background would result in a flat line till higher fields and would adopt a linear slope at higher fields). To disregard any spurious effect from the fitting procedure, the numerical derivative

of the measured non-local voltage for an applied out-of-plane field is shown in Fig. S18 d. We observe a sharp change in the derivative corresponding to the spin flip field of $CrPS_4$. Furthermore, if the background signal were only due to the Nernst effect, the derivative is expected to be a constant while we observe a non-linear background.

VIII.C. Non-Local Second Harmonic response of Pt

The local second harmonic response across Pt, namely the local SSE is reported in SI VII. We note that the measured non-local second harmonic response across Pt is consistent with the non-local SSE (as shown in Fig. S19), similar to the earlier reports of local SSE across Pt on $CrPS_4$ [8, 9].

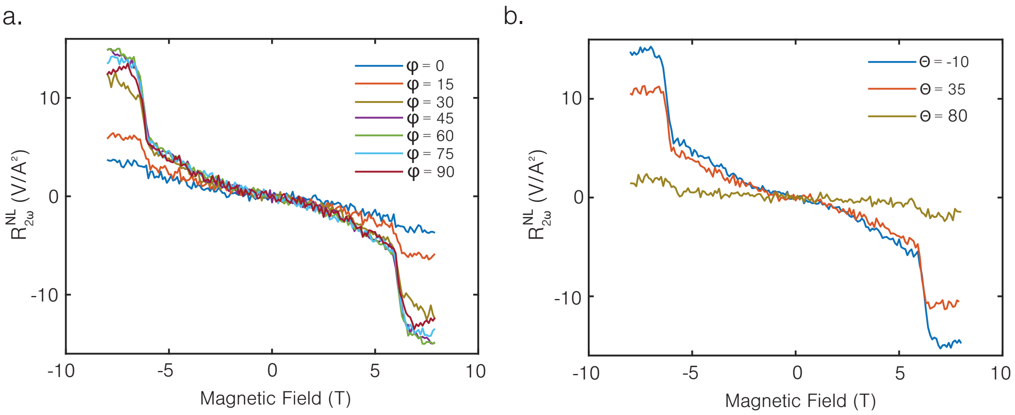

FIG. S19: The non-local second harmonic responses across Pt for various a) out-of-plane field angles, b) in-plane angles (at 25K and $i_{WTe_2} = 100\ \mu A$).

However, we note that while injecting with WTe_2 the MR of WTe_2 being extremely large, results in a thermal gradient that is field dependent (as discussed in section SI VIII) due to which in Fig. S19 a we observe a slight shift in the trend of the NL-SSE as a function of the out-of-plane field angle.

IX. COMPARISON OF INTERFACE SPIN CONVERSION EFFICIENCY

Magnon spin transport through $CrPS_4$ has been previously quantified using the current transfer efficiency η, as reported in Ref. [9], to determine the magnon conductivity and magnon spin diffusion length. Following the approach in Ref. [15], a charge current I in the platinum injector generates a transverse spin current via the spin Hall effect (SHE), resulting in a spin accumulation μ_s at the Pt/$CrPS_4$ interface. The injector conversion coefficient is defined as $\eta_{\rm inj} = \mu_s/(eI)$, where e is the elementary charge, and $\eta_{\rm inj}$ depends on the material parameters and geometry of the Pt strip. The spin accumulation generated in the injector via SHE is given by:

$$\mu_s = 2\mathrm{eI}\theta_{Pt}\frac{l_s}{\sigma_e t w}\tanh(\frac{t}{2l_s}), \tag{14}$$

where e,t,w,θ_{Pt}, l_s and σ_e are the electronic charge, thickness of the Pt strip, width of the Pt strip, spin Hall angle, spin diffusion length and the electrical conductivity of Pt [15]. The exchange interaction at the interface induces a magnon chemical potential μ_m in $CrPS_4$. Assuming that the interfacial spin resistance is negligible (*i.e.* that it is the bulk magnon transport parameters σ_m, λ_m govern the non-local resistance measured [16]), which results in $\mu_m \approx \mu_s$. Thus by assuming a large spin mixing conductance at the Pt/$CrPS_4$ interface ($\eta_{\rm inj} = \mu_s/(eI)$), the measured non-local resistance (normalized to the length of the injector/detector strip) is then given by:

$$R_{NL} = \frac{\sigma_m t_{CPS}\eta_{Pt}\eta_{WTe_2}}{\lambda_m}\mathrm{csch}(\frac{d}{\lambda_m}), \tag{15}$$

where R_{NL}, σ_m, t_{CPS}, η_{Pt/WTe_2}, d and λ_m correspond to the non-local resistance normalized with respect to the length of the injector/detector, magnon conductivity, the thickness of $CrPS_4$, the spin accumulation conversion coefficients of Pt and WTe_2, the distance between Pt and WTe_2 and the magnon diffusion length [9]. Intuitively, from Eqn. 15, in the limit $\lambda_m \to \infty$, corresponding to ballistic magnon transport, the quantity $\sigma_m t_{\rm CPS}\eta_{\rm Pt}\eta_{\rm WTe_2}$ represents the expected non-local resistance. This expression reflects the combined influence of the magnon conductivity of the medium and the charge-to-spin conversion efficiencies of the injector and detector.

To extract $\eta_{\rm WTe_2}$, we use reported values of the magnon diffusion length $\lambda_m = 696\,\mathrm{nm}$ and magnon conductivity $\sigma_m = 9.77 \times 10^3\,\mathrm{Sm^{-1}}$ at 25 K and 7.75 T from Ref. [9]. For the Pt strip used (12 nm thick, 650 nm wide), we estimate $\eta_{\rm Pt} = 0.03\,\Omega$. Assuming the same magnon conductivity and diffusion length for out-of-plane and in-plane polarized magnon spins, we extract the spin accumulation conversion coefficient of WTe_2 as $0.1211\,\Omega$ for out-of-plane polarized magnon spins and $0.0312\,\Omega$ for in-plane polarized magnon spins.

The relative injection efficiencies reported in the main text are referenced to standard Pt electrodes (7 nm thick, 300 nm wide), for which $\eta_{\rm Pt} = 0.07\,\Omega$. We note that the assumption of large spin mixing conductance is applied only at the Pt/$CrPS_4$ interface, allowing for the substitution of $\eta_{\rm inj} = \mu_s/(eI)$. No such assumption is made for the WTe_2/$CrPS_4$ interface. Therefore, the reported $\eta_{\rm WTe_2}$ in the main text reflects both the charge-to-spin conversion efficiency of WTe_2 and the interfacial spin mixing conductance, which we refer to as the effective charge-to-spin interconversion efficiency.

X. COMPARISON WITH DEVICE S1

The sample fabrication of device S1 is similar to the sample discussed in the main text, with the exception of the order of stacking and platinum deposition. For sample S1, the dry transfer of WTe_2 and etching into strips were performed before the DC sputtering of platinum (∼9 nm). The thickness of $CrPS_4$ and WTe_2 flakes measured by AFM were 130 nm and 17 nm respectively. The length of the strips and the distance between the strips measured by SEM was 38.2 μm and 255 nm respectively. The width of the platinum and the WTe_2 strips were 650 nm and 900 nm respectively. Other sample fabrication and measurement steps were maintained the same.

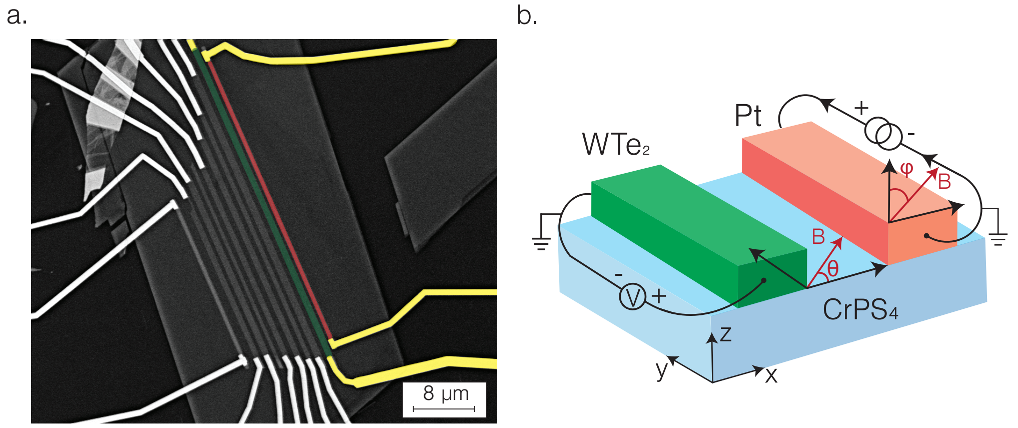

FIG. S20: a) Scanning electron micrograph (false-colored) of Device S1 and b) illustration of the non-local geometry of Device S1.

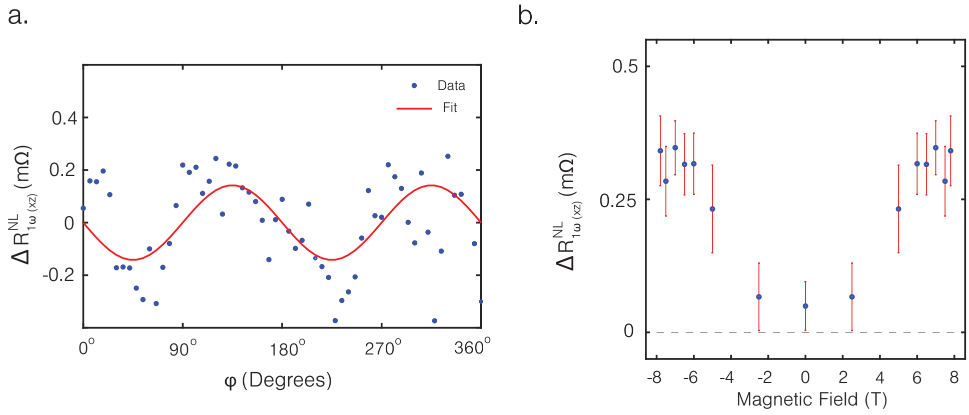

FIG. S21: The a) non-local resistance response as function of out-of-plane angular rotations at 7.8T and b) the amplitude of the non-local resistance as a function of the applied magnetic field (Temp = 10K, i_{Pt} = 100 μA).

Fig. S21 shows the the non-local resistance modulation and the field dependency of the amplitude for out-of-plane angular rotations (extraction of the out-of-plane component was achieved using the same protocol as described in section SI V). We note that the measured non-local resistance in the out-of-plane angular rotations is reproducible across different devices. In addition to the rapid onset in the non-local resistance corresponding to the spin-flip field of $CrPS_4$, we further see a saturation in the non-local resistance at higher fields similar to that reported in Ref. [9]. The observation of saturating non-local resistance with higher magnetic fields show that the spin injection efficiency of WTe_2 is not affected by the applied magnetic field. Within the noise limit of our measurements, we could not detect a non-local signal for in-plane angular rotations.

XI. CROSS-SECTIONAL TRANSMISSION ELECTRON MICROSCOPE

To further understand the quality of the interfaces and the underlying cause behind the difference in the thickness of Pt recorded on Si/SiO_2 and $CrPS_4$ (as mentioned in section SI I), the devices used in this work were further analyzed by means of Scanning Transmission Electron Microscopy (STEM).

Focused Ion Beam (FIB) sample preparation: The FEI Helios G5 CX dual-beam SEM-FIB (scanning electron microscope-focused ion beam) was used to prepare electron-transparent samples. Before adding the protective Pt layer, protective carbon (C) and platinum (Pt) were deposited on the film using Electron Beam Induced Deposition (EBID). Cross-sectional chunks (dimensions: 15 x 2.0 x 5 μm^3) were made and transferred using an EasyLiftTM needle to the copper half-grid. The sample was then thinned down to 80-100 nm thickness using standard Ga-beam processing at 30 kV with an opening of a certain window (6.0 x 5.0 μm^2). The remaining chunk is left thick enough to have a rigid frame to minimize the bending and stress release in the e-transparent window. Finally, several low kV cleaning steps (5 and 2 kV) were used to clean the side surfaces of the lamellae.

Scanning/Transmission Electron Microscopy (STEM): The FIB-prepared and loaded TEM grid was transferred immediately to the TEM column using a dedicated double tilt TEM holder optimized to collect x-rays in the TEM. The microstructure of samples were examined with a double-corrected (probe and image correctors) and monochromated Themis Z scanning transmission electron microscope (Thermo Fisher Scientific) operating at 300 kV. The STEM images were acquired through HAADF (high-angle annular dark-field) mode and simultaneously integrated differential phase contrast (iDPC) mode. The beam convergence angle was $\sim$18 mrad and a probe current of 15 pA was used for STEM imaging. Energy dispersive X-ray spectroscopy (STEM EDS maps) results were achieved with a Dual X EDS system (Bruker), using two large area detectors in total capturing 1.76 steradian with a probe current of 50 pA for more than 2 hours. Data acquisition and analysis were performed using Velox software.

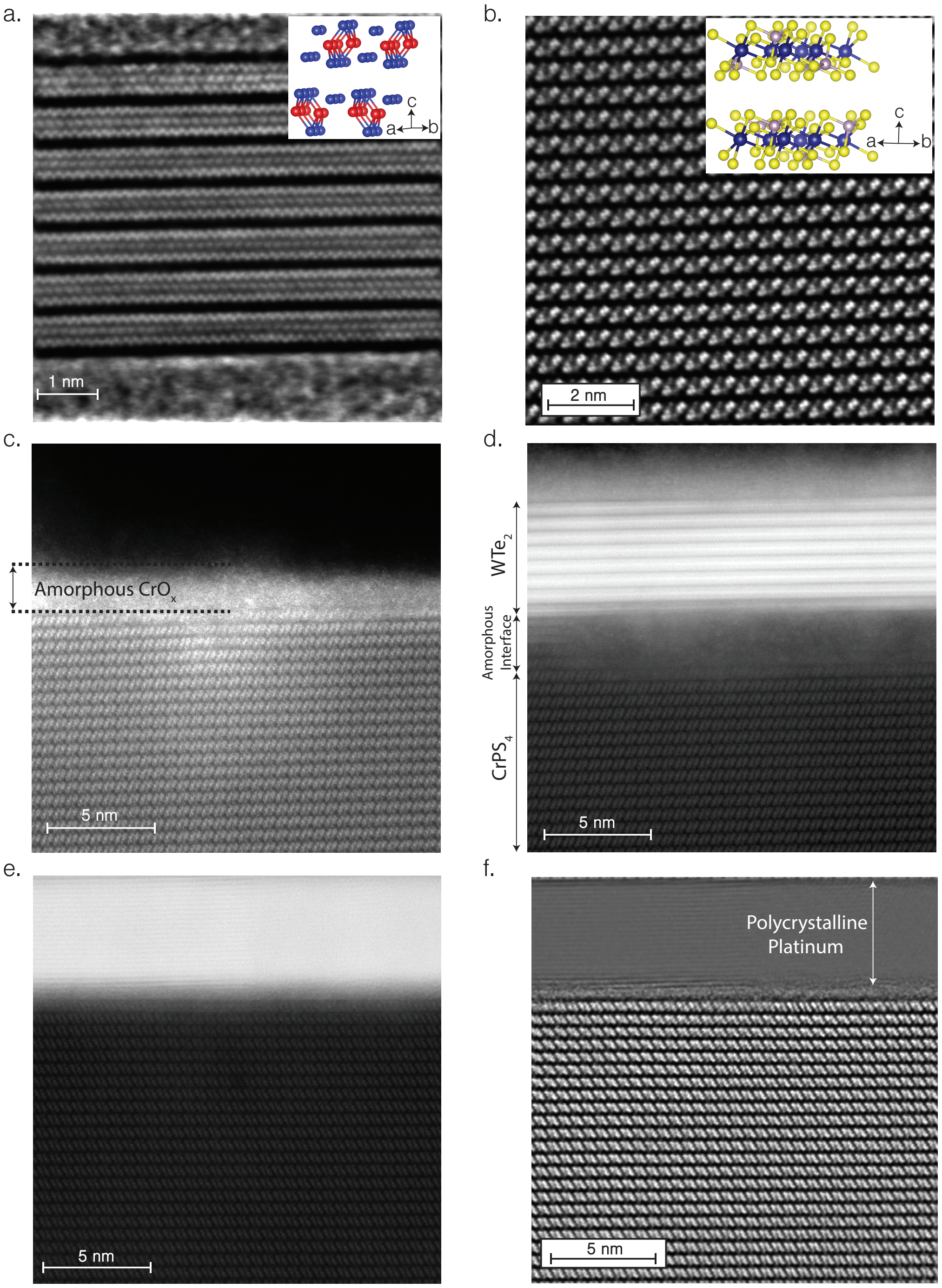

FIG. S22: iDPC-STEM images of a) WTe_2, b) $CrPS_4$, STEM images of c) $CrPS_4$ exfoliated under ambient conditions, d) the $WTe_2/CrPS_4$ interface of device S1, e) the Pt/$CrPS_4$ interface of the device M1 and f) iDPC-STEM images of Pt/$CrPS_4$ interface.

Fig. S22a,b shows the integrated Differential Phase Contrast STEM (iDPC-STEM) images of WTe_2 and $CrPS_4$. From Fig. S22c, we observe that $CrPS_4$ exfoliated under ambient conditions oxidize to form a capping layer of amorphous CrO_x, about 2-3 nm thick, that passivates the surface and protects the rest of the bulk from oxidation, similar to that observed with WTe_2 [17]. Fig. S22d,e show the STEM images of the $WTe_2/CrPS_4$ and Pt/$CrPS_4$ interfaces. At the $WTe_2/CrPS_4$, we observe an amorphous layer of about 2-5 nm. We attribute this amorphous layer to the interaction between WTe_2 (upon dry transfer) and the CrO_x layer that was present at the surface of $CrPS_4$.

However, surprisingly, we do not observe this CrO_x layer at the interface between Pt and $CrPS_4$. The effect of DC sputtering involving highly energetic particles (for instance, sputtered atoms off the target, reflected Ar atoms) onto two-dimensional materials has been investigated for the potential damage they can cause to the surface [18–20]. One possible explanation for the absence of the amorphous layer could be that the process of DC sputtering Pt on the $CrPS_4$ surface is abrasive and ends up removing the initial oxide layer that was present which could also explain the AFM results in section SI I. Despite the absence of the CrO_x layer, we observe an amorphous interface between Pt and $CrPS_4$ of about 1nm thickness. To further understand the interface, the profile of the chemical composition across the interface was acquired in the STEM mode by means of an energy-dispersive X-ray (EDX) spectroscopy signal.

The STEM image and the corresponding chemical (EDX) profile obtained across the various interfaces are shown in Fig. S23. Similar to the report of the interface between Pt and $MnPSe_3$ in Ref. [21] interface, we observe an intermediate PtS_x layer of about 1 nm thick between the deposited polycrystalline Pt layer and the $CrPS_4$ flake (although the exact chemical composition is hard to extract, we do not observe an oxide at the interface).

At the $WTe_2/CrPS_4$ interface, we observe that the interface is predominantly an oxide. We note two distinct oxide layers: the first a WO_x layer and the latter a TeO_x layer (where TeO_2 is the known stable oxide of tellurium). Noticeably from Fig. S23c, we see that the TeO_x lies below the native surface of isolated $CrPS_4$ suggesting the atomic diffusion of tellurium atoms into the CrO_x layer. Atomic diffusion of tellurium atoms has been widely investigated across a wide range of interfaces [22–24].

As the spin-injection efficiency is highly sensitive to the interfacial quality, minimizing disorder at the interface is essential. In our experiments, even with an approximately 4 nm thick amorphous barrier between WTe_2 and $CrPS_4$, we still observe transport signatures consistent with magnon-mediated spin currents reported with Pt [8, 9]. This suggests that while our current interface is sufficient to inject/detect magnon spins, reducing or eliminating the barrier could further improve spin-injection efficiency.

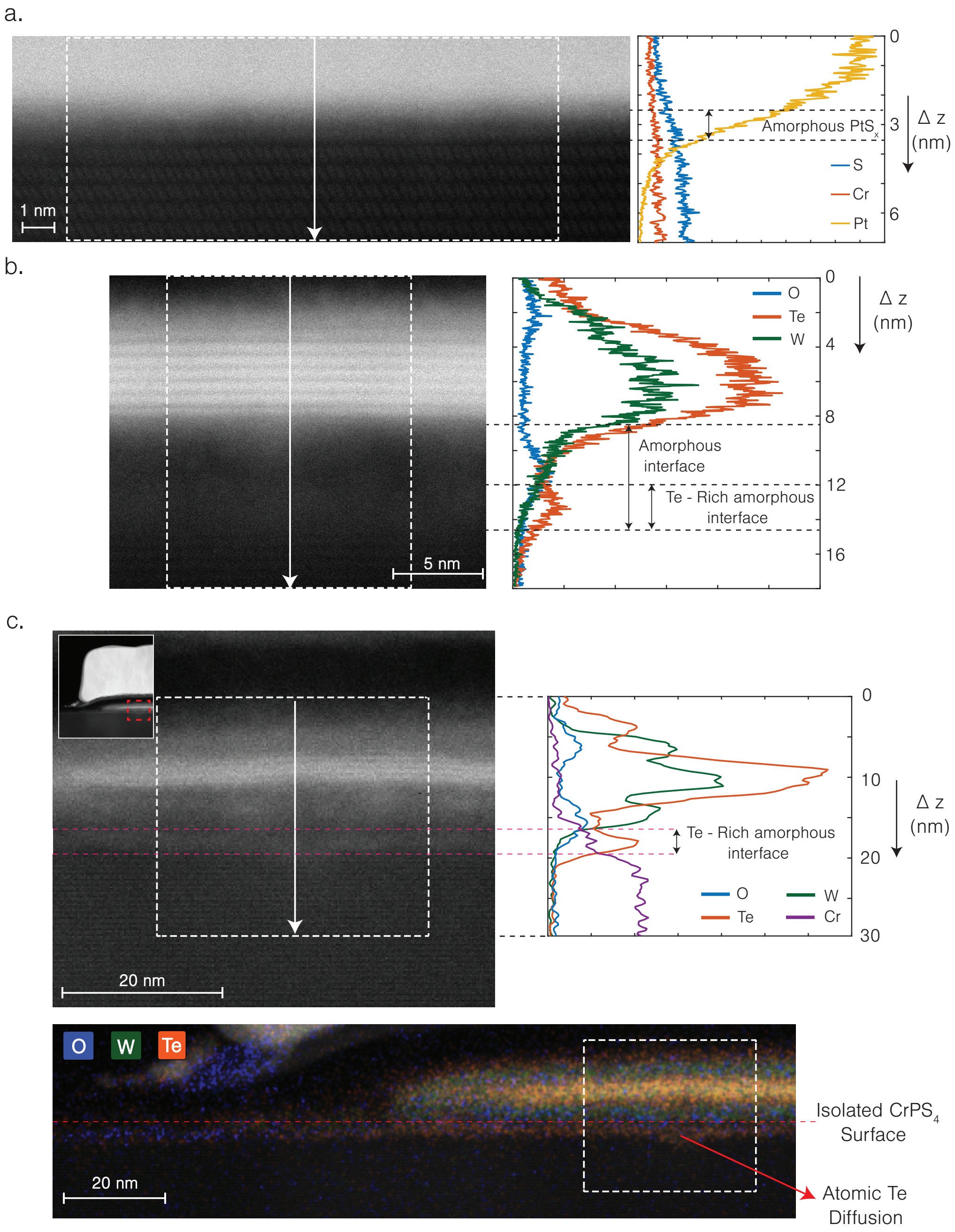

FIG. S23: The STEM image and the corresponding EDX profile for the analysis of the chemical composition of the a) Pt/$CrPS_4$ interface from the device M1, b) WTe_2/$CrPS_4$ interface of device S1 and c) WTe_2/$CrPS_4$ interface of device M1 (Inset shows the entire HAADF-STEM image of the EDX scan).

XII. COMPARISON WITH DEVICE S2

The sample fabrication of device S2 is similar to device S1 mentioned earlier. In comparison to the asymmetric injector-detector combination of Pt/WTe_2, in device S2, both the injector and detector electrodes were WTe_2 in an all two-dimensional heterostructure.

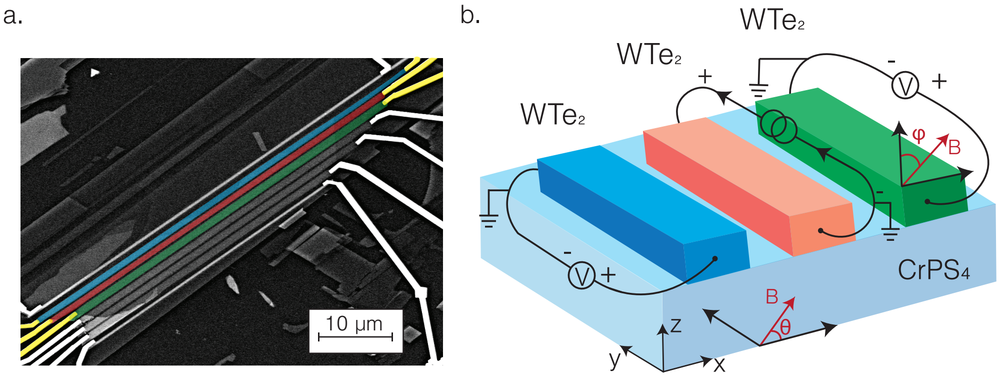

FIG. S24: a) Scanning electron micrograph (false-colored) of Device S2 and b) illustration of the non-local geometry of Device S2.

Fig. S21 shows the amplitude of the non-local response modulation and the magnetic field dependence of the amplitude for out-of-plane angular rotations measured across the WTe_2 detectors on either side of the injector. Now, given that both the injector and the detector electrodes exhibit a large MR, the modulation of the Y-Component is too large and thus its parasitic leakage into the X-component, making the extraction of the out-of-plane polarized magnon spin signal as described in section SI V difficult.

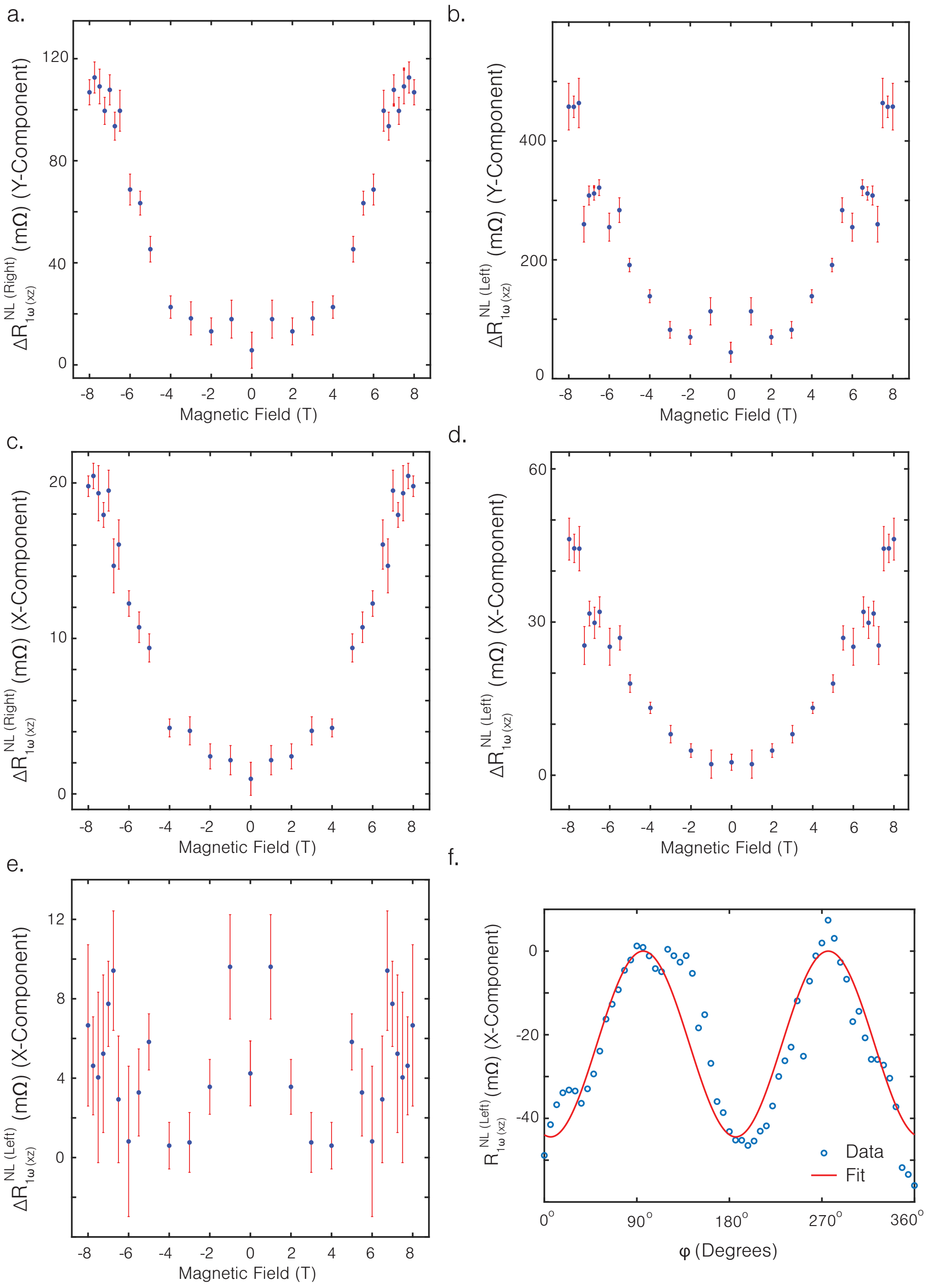

FIG. S25: The amplitude of the non-local resistance modulations as function of applied field for a) out-of-phase component and b) in-phase component of the left detector; c) out-of-phase component and d) in-phase component of the right detector; e) the amplitude extracted using the protocol described in section SI V and f) the non-local resistance modulation of the left detector (Temp = 25K, $i_{WTe_2} = 50\ \mu A$).

Although we are unable to extract the magnonic transport signal from the all two-dimensional sample, we emphasize that WTe_2 serves as a promising platform among van der Waals materials for excitation and detection of magnon spin current in two-dimensional magnets with lower saturation fields where the modulation of the Y-component might not be as sizable.

References

[1] T. Liu, A. Roy, J. Hidding, H. Jafari, D. K. De Wal, J. Sławińska, M. H. Guimarães, and B. J. Van Wees, Physical Review B **108**, 165407 (2023).

[2] S. Kim, J. Lee, C. Lee, and S. Ryu, The Journal of Physical Chemistry C **125**, 2691 (2021).

[3] J. Lee, T. Y. Ko, J. H. Kim, H. Bark, B. Kang, S.-G. Jung, T. Park, Z. Lee, S. Ryu, and C. Lee, ACS nano **11**, 10935 (2017).

[4] D. MacNeill, G. Stiehl, M. Guimaraes, R. Buhrman, J. Park, and D. Ralph, Nature Physics **13**, 300 (2017).

[5] M. Kim, S. Han, J. H. Kim, J.-U. Lee, Z. Lee, and H. Cheong, 2D Materials **3**, 034004 (2016).

[6] X.-G. Ye, H. Liu, P.-F. Zhu, W.-Z. Xu, S. A. Yang, N. Shang, K. Liu, and Z.-M. Liao, Physical Review Letters **130**, 016301 (2023).

[7] F. Wu, M. Gibertini, K. Watanabe, T. Taniguchi, I. Gutiérrez-Lezama, N. Ubrig, and A. F. Morpurgo, Advanced Materials **35**, 2211653 (2023).

[8] D. K. De Wal, A. Iwens, T. Liu, P. Tang, G. E. Bauer, and B. J. Van Wees, Physical Review B **107**, L180403 (2023).

[9] D. K. de Wal, M. Zohaib, and B. J. van Wees, Physical Review B **110**, 174440 (2024).

[10] B. Feng, Y.-H. Chan, Y. Feng, R.-Y. Liu, M.-Y. Chou, K. Kuroda, K. Yaji, A. Harasawa, P. Moras, A. Barinov, et al., Physical Review B **94**, 195134 (2016).

[11] L. Cornelissen, J. Liu, R. Duine, J. B. Youssef, and B. Van Wees, Nature Physics **11**, 1022 (2015).

[12] L. Thoutam, Y. Wang, Z. Xiao, S. Das, A. Luican-Mayer, R. Divan, G. Crabtree, and W. Kwok, Physical review letters **115**, 046602 (2015).

[13] Y. Pan, B. He, T. Helm, D. Chen, W. Schnelle, and C. Felser, Nature communications **13**, 3909 (2022).

[14] M. Behnami, M. Gillig, A. Moghaddam, D. Efremov, G. Shipunov, B. Piening, I. Morozov, S. Aswartham, J. Dufouleur, K. Ochkan, et al., Physical Review Research **7**, 023009 (2025).

[15] X.-Y. Wei, O. A. Santos, C. S. Lusero, G. Bauer, J. Ben Youssef, and B. Van Wees, Nature Materials **21**, 1352 (2022).

[16] L. J. Cornelissen, K. J. Peters, G. E. Bauer, R. A. Duine, and B. J. van Wees, Physical Review B **94**, 014412 (2016).

[17] F. Ye, J. Lee, J. Hu, Z. Mao, J. Wei, and P. X.-L. Feng, arXiv preprint arXiv:1608.00097 (2016).

[18] F. O. Johansson, P. Ahlberg, U. Jansson, S.-L. Zhang, A. Lindblad, and T. Nyberg, Applied Physics Letters **110** (2017).

[19] M. Saifur Rahman, A. D. Agyapong, and S. E. Mohney, Journal of Applied Physics **136** (2024).

[20] R. Hertwig, S. Nishiwaki, A. N. Tiwari, and R. Carron, Solar RRL **6**, 2200268 (2022).

[21] S. Catalano, J. M. Gomez-Perez, M. X. Aguilar-Pujol, A. Chuvilin, M. Gobbi, L. E. Hueso, and F. Casanova, ACS Applied Materials & Interfaces **14**, 8598 (2022).

[22] Q. Gong, H. Jiang, J. Perrin-Toinin, M. Peterlechner, M. Putero, A. Portavoce, S. Divinski, and G. Wilde, Acta Materialia **257**, 119146 (2023).

[23] G. Sosso, J. Behler, and M. Bernasconi, physica status solidi (a) **213**, 329 (2016).

[24] T.-Y. Yang, I.-M. Park, B.-J. Kim, and Y.-C. Joo, Applied Physics Letters **95** (2009).
